# Ionospheric TEC anomalies analysis and prediction during six volcanic eruptions using a Hybrid ML-DL model and comparison with the AR/MLR Models

**DOI:** 10.1371/journal.pone.0354386

**Published:** 2026-07-24

**Authors:** R. Mukesh, S. Logesh, Sarat C. Dass, G. Cynthia, M. Sudhandra, K. Sivaprabha, T. Annu Regha, S. Kiruthiga

**Affiliations:** 1 Department of ECE, Saranathan College of Engineering, Trichy, India; 2 School of Mathematical and Computer Sciences, Heriot-Watt University Malaysia, Putrajaya, Malaysia; King Abdullah University of Science and Technology, SAUDI ARABIA

## Abstract

Eruption of volcanoes is associated with the emission of great energy through the propagation of atmospheric waves up into space. These waves cause significant disturbances in the ionosphere, a region that contains a large number of ions and electrons and is important in providing communications through radio waves and satellites. Disturbances may cause the formation of Equatorial Plasma Bubbles and low-density pockets, leading to signal distortion, time delays, and sometimes, loss of signals in satellites. The Global Positioning System (GPS) becomes inaccurate due to these disturbances since GPS uses information on the positions of user locations. Satellite-based services like the internet and telephony may be affected by the disturbances in the ionosphere caused by a high number of electrons. Prediction of these disturbances makes it possible to plan ways of mitigating against them during future eruptions. Total Electron Content (TEC) data used in this research were mainly obtained from the BAKO station in Indonesia, in addition to other latitudes, in order to prove the universality of the model. The Hybrid Machine Learning and Deep Learning (ML-DL) Model, which is a combination of Light Gradient Boosting Machine (LightGBM) and Long Short-Term Memory networks (LSTM) algorithms, was designed based on a weighted average and applied in forecasting TEC values. Performance of the model was compared with other independent models, i.e., individual LightGBM and LSTM, as well as the conventional Autoregressive (AR) and Multiple Linear Regression (MLR) models. Four evaluation measures were used in evaluating the performance of models. This study aims to assess the performance of the Hybrid ML-DL Model in predicting TEC during ionospheric disturbances caused by volcanic eruptions. TEC prediction was done in six major eruptions, including Mt. Kelud (2014), Mt. Sinabung (2016), Mt. Semeru (2021), Mt. Ruang (2024), Mt. Etna (2013), and Mt. La Soufriere (2021). The Hybrid ML-DL Model consistently performs better than the individual LightGBM and LSTM models and the traditional AR and MLR models in predicting TEC values. For example, during the Mt. Ruang eruption, which was the highest eruption disturbance analyzed with a maximum disturbance of 100–110 TECU between April 16th and May 7th, 2024, the Hybrid ML-DL Model scored an RMSE of 2.841 TECU, which was much lower than RMSE values of LightGBM (3.484 TECU), LSTM (5.084 TECU), MLR (5.345 TECU) and AR Model (6.285 TECU). The Hybrid ML-DL Model showed the least value in three performance evaluation criteria, including NRMSE (0.030), MBD (0.546 TECU), and RLE (0.069) among other models.

## 1. Introduction

Volcanic Eruptions are powerful natural events that release immeasurable energy. When a volcano breaks out, intense atmospheric gravity waves are generated. These waves are caused by the vertical air shift in air caused by eruptions, and generate vibrations within the Earth’s atmosphere. When these gravitational waves hit, they eventually reach the upper layers of the atmosphere, especially the ionosphere. The ionosphere is the region of the Earth’s atmosphere, ionized by solar and cosmic radiation, and contains highly concentrated ions and free electrons. This layer plays an important role in the spread of radio waves and satellite communications. After reaching the ionosphere, gravitational waves cause obstacles in this area. These disorders can lead to the formation of Equatorial Plasma Bubbles (EPBs), a local region with significantly lower plasma density compared to the surrounding area. An EPB is essentially a pocket or cavity within the ionosphere. Irregularities caused by EPBs in the ionosphere can scatter radio waves and break. This scattering effect degrades the quality of the signal, weakening or distorting it. In severe cases, it can even block radio wave transmission completely. For example, GPS signals (Global Positioning Systems) can be affected, leading to inaccuracy when determining positions.

Communication satellites used for radio, internet and telephone services can experience signal degradation, leading to a decline in the quality and reliability of these services. Scientists can improve weather prediction by examining the interactions between atmospheric gravitational waves, ionospheres, and EPBs. TEC forecasting is the most essential process for predicting the ionospheric behaviour for space communication. Today, the current algorithms face several challenges, as most of the algorithms focus on solar and geomagnetic activity; often, they don’t look for disaster events like volcanic eruptions, tsunamis, and severe weather conditions, which also generate ionospheric disturbances. Here, the large-scale events are easily measured, whereas small-scale irregularities like EPBs make it difficult to capture, which leads to a reduction in forecasting accuracy in equatorial and low-latitude regions. Many algorithms perform well in near-term forecasts but struggle with the medium and long-term forecasts.

The limitations of existing TEC prediction algorithms and methods are detailed below as:

Current TEC forecast methods, ranging from empirical and physics-based models to contemporary machine learning methods, have all failed to achieve adequate accuracy under perturbed or extreme space weather conditions. Methods which are based on Global Ionospheric Maps (GIMs), spherical harmonic coefficients, or solar and geomagnetic indices such as Dst and EUV flux are good at capturing diurnal and seasonal patterns under quiet times but deteriorate during geomagnetic storms or sudden solar variability. Research has revealed that deep learning models of spherical harmonic coefficients exhibit a significant rise in RMSE during storm periods, denoting a sudden decrease in reliability [1]. Likewise, models that were trained mostly on quiet periods do not generalize well under extreme events, and GNSS-based region-specific models demonstrate good performance only in densely instrumented regions, and error grows over weakly observed regions like oceans and polar regions [2].

Another significant limitation is the tradeoff between forecast lead time and accuracy. As the prediction horizon increases, errors accumulate with some uncertainties on both input parameters and model structure, limiting reliable forecasts to short timescales. Physics-based methods need precise initial and boundary conditions, which are not typically available during rapidly evolving solar activity. In contrast, more sophisticated deep learning architectures such as ConvLSTM and spatio-temporal GNNs, while able to learn intricate TEC dynamics, are computationally intensive, hard to interpret, and data-lag or data-gap sensitive. By extension, operational TEC forecasting continues to suffer from limitations in demonstrating consistent accuracy, real-time usability, and physical interpretability, particularly during geomagnetic disturbances [3].

This study determines the comparative assessment usage of Hybrid Machine Learning and Deep Learning (ML-DL) i.e., a combination of both LightGBM and LSTM for forecasting TEC during Volcanic Eruptions around the world. Table 1 shows the list of Volcanic Eruptions at different Mountains across different years in the world.

**Table 1:** List of volcanic eruptions that occurred at different mountains in the world.

Mountains chosen for analysis	Volcanic Eruption Occurred Periods
Mt.Kelud	13.02.2014–21.02.2014
Mt.Sinabung	18.05.2016–24.05.2016
Mt.Semeru	04.12.2021–06.12.2021
Mt.Ruang	16.04.2024–07.05.2024
Mt.Etna	15.11.2013,16.11.2013,17.11.2013, 22.11.2013,28.11.2013 and 12.12.2013
Mt. La Soufriere	09.04.2021–22.04.2021

To demonstrate how our model forecasts TEC over a range of eruption intensities, we chose to analyze specific events. We primarily picked the major impact days during every eruption. We saw that whereas some eruptions were only 2 days long, others initiated high warning levels that lasted for more than 3 years. Evaluations are conducted against LightGBM and LSTM, as well as AR (Auto Regression) and MLR (Multiple Linear Regression) as reference models, revealing an advantage of using sophisticated machine learning methods, which are increasingly used to improve forecasting precision under extreme space weather conditions. Researchers are incorporating real-time GNSS signals into dynamic observations of space weather in order to develop improved predictive models, which more accurately model ionospheric responses. Combining data sources allows for a more accurate estimation of Total Electron Content (TEC), which is important to the integrity of satellite-based navigation systems. Particularly in equatorial and low-latitudinal areas—where the frequency of disturbances is higher—these optimized models assist in providing even performance. The method enhances the robustness of GPS and other satellite services, even when intense disruption is occurring in the atmosphere.

Chen et. al., (2025) have described the integration of ground-based GNSS and space-based COSMIC-2 occultation data and GIM for analysis. Initially, the GNSS ionospheric inversion method was employed, but it showed certain limitations in the spatial distribution of ground-based GNSS. In order to overcome this defect, the above-mentioned joint observation has been used to further improve the spatio-temporal distribution of ionospheric monitoring. The experiments depicted in the paper showed that the average of TEC of GIM data was found to drop by 7.5 TECu, which aligned with the data obtained from the TONG station. The data was inversely proportional to the distance between the station and the volcanic site. From this, the observation was ruled out to prove the consistency in the derived model [4].

Feng et.al., (2023) studied the ionospheric anomalies before the submarine volcanic eruption of Hunga Tonga-Hunga Haapai on 15 January. For this GNSS station’s observational data, Global ionospheric maps and electron density of FORMOSAT-7/COSMIC-2 occultation were used. Using this, a negative TEC anomaly was detected by three GNSS stations and GIMs on January 5 near the epicentre. The equatorial anomaly wave peaks moved towards the South Pole by observing the latitude-time-TEC variations sequentially. From their data analysis, the TONG station shows a decrease in the peak of the diurnal ionosphere by nearly 10 TECU on 4th January [5].

Shinagawa & Miyoshi (2024) studied an axisymmetric three-dimensional non-hydrostatic atmospheric model that has been developed to perform simulation studies of the volcano of Hunga Tonga-Hunga Haapai in Tonga that erupted on January 15. The model was then coupled with GAIA. Even though multiple models have been devised, the phenomenon related to its resultant acoustic waves has not been defined clearly. The simulation model can be used to analyze many kinds of atmospheric waves generated. Some examples of such waves are acoustic waves, gravity waves, Lamb waves, Pekeris waves and TID concentrically propagated from the eruption site. After the initial large oscillations, acoustic resonance appears in the thermosphere of about 4 min [6]. Pandey et. al., (2024) used Global Positioning System data to understand and analyze pressure waves and propagation of long-period ionospheric disturbances of the Tonga-Honga volcanic eruption on January 2022. The strong ionospheric anomalies westward propagation was also inferred in this publication. Most observational perturbances were concentrated near the polar regions. The irregularities of pressure waves propagated with a velocity of 320m/s was an important result demonstrated. From the observance, the first estimation of the amplitude decay of the CVIDs is identified as potentially used for modelling the propagation of such disturbances. Surface pressure perturbations were identified 36 hours before the occurrence [7].

Pandara et. al., (2021) use data from dual-frequency Geographic Positioning Systems observations. Lokon volcano is found to be one of the most active volcanoes in Indonesia. Before this research, no other papers have carried out research on TEC distribution related to Lokon eruptions. This research combined RSAM data and the GPS TEC data to determine the effect of eruption activities on the ionosphere. This research stressed that volcanic eruptions and seismic activities can trigger the gravitational and acoustic waves upward into the atmosphere [8]. Carter et. al., (2023) analyzed the impact of the Hunga Tonga Volcanic eruption on GPS Precise Point Positioning. This was done through observing the volcano’s ionospheric disturbances. A combination of GNSS receivers was used to explore the volcanic eruption’s impact. An increase in convergence times has been found to be caused by a super Equatorial Plasma Bubble (EPB) upon further studies. The structure was found to be 42 TEC deep and travelling at 30 m/s. The ionospheric variability that impacts real-world applications served a greater deal [9]. Qiu et. al., (2023) studied the response of the volcanic eruption of the Tonga volcano. These observations were specifically concentrated from the low-latitude station of the Meridian Project at Fuke, Hainan. The volcanic eruption caused anomalies that were identified in plasma drift during the main eruption. A sudden increase and inversion of the plasma drift velocity, followed by a large fluctuation in drift velocity, were observed. These abnormalities were analyzed to conclude that it was the response of the low-latitude ionosphere to the Tonga volcanic eruption. Possible mechanisms that can be ruled out for these effects on ionospheric plasma drift were also concluded [10]. He et. al., (2022) elaborate on the finding of the occurrence of the eruption during a moderate geometric storm. As a result, the depletion was found to be a local negative effect of the storm. This analysis was proposed for the first time in this research. Also, the relative contributions of the eruption and the storm are validated by means of measurements. Parameters considered are GNSS-derived vertical Total Electron Content, O/N2 ratio by TIMED/GUVI, ion density and temperature by ICON/IVM. Analysis by using simulation was also carried out in his work by using the Thermosphere Ionosphere Electrodynamics General Circulation Model (TIEGCM). Shortly after the volcanic eruption, VTEC reduced by 80% to 95% [11].

Flores et. al., (2024) had produced one-dimensional satellite data over the geographic area around the Tonga-Hunga Haapai volcanic eruption. Ionospheric Total Electron Content (TEC) and Global Navigation Satellite Systems ground receivers were used for data collection. The paper identifies the ionospheric anomalies travelling from southeast to northwest. They maintain a constant speed of 0.3 km/sec. They were identified as TIDs with several wavefronts. For further improvements in observation, maximizing the use of GPS data from many dense and widely distributed stations is encouraged in this paper [12]. Guerra et. al., (2024) aimed to compare and understand different TEC detrending techniques. It also validates how their settings cause an impact in retrieving such parameters. Fast Iterative Filtering and the Savitzky-Golay filter are highlighted as the most reliable techniques. They are proven in comparison with moving average, multi-order numerical difference, polynomial detrending and Finite Impulse response filtering techniques. Furthermore, the impact of IPP height and elevation cut-off angle is identified to have a high impact on the retrieval period. The study also proposes techniques to accurately estimate NRT (near real-time) TIDs classification and detection [13].

Zhang et. al., (2022) gave a whole picture of ionospheric disturbances caused by the Tonga volcanic eruption. The observation was undertaken over a period of 4 days at a minimum. From the observations, TIDs were found radially outbound and inbound along entire Great-Circle loci. Their characteristics were identified with a 300−350 m/s speed and 500−1,000 km horizontal wavelength for front shocks. The propagation is found to be consistent with the Lamb waves effect that travels at the speed of light (3x10^8 m/s). The study also provides substantial first evidence for long-duration imprints [14]. Martire et. al., (2022) provided a near-real-time ionospheric monitoring software named GUARDIAN. It’s proposed to establish a natural hazards warning. It makes use of TEC time series data to explore TEC perturbations that are created due to natural and anthropogenic events on Earth. They facilitate the automatic detection and characterization of potential natural hazards. The software focuses on collecting GNSS measurements in time series format, computing TEC and publishing them on a public website. Until now, the GUARDIAN system uses more than 70 GNSS ground stations for its operation and monitors – GNSS constellations [15].

Cahyadi et. al., (2024) were undertaken for the ideal purpose of using GNSS TEC to detect TIDs after the eruption of Hunga Tonga-Hunga Haapai. Two types of tsunamis were caused after eruption. The first one was generated by atmospheric waves and the later by the eruption. From the analysis, a moderate correlation between TID amplitude and tsunami wave height models was found from tide gauge stations in New Zealand and Australia. 3D structure of TIDs was tried using 3D tomography. Concentric directivity of the atmospheric wave generated the first TID was observed by means of a tomogram [16].

Shinbori et. al., (2022) highlighted the characteristics of electromagnetic conjugacy of displacing ionospheric disturbances after the eruption of the Hunga Tonga-Hunga Haapai volcano. The date of observation was found immediately after January 15 2022. Also, the thermal infrared grid data has been used to observe lower atmospheric disturbances. The data was ascertained with high resolution by the Himawari satellite in Lamb mode. One of the notable findings is that two unique travelling ionospheric disturbances were identified in the Southern Hemisphere. The flow of plasma changing its direction to northward with an amplitude and period of 100-110m/s and 36–38 mins is notably stated in this research [17]. Sun et. al., (2022) stressed the impact of various waves produced by the Hunga-Tonga volcano eruption in January 2022. The generation of perturbation waves, as well as the equatorial plasma bubbles (EPB) over the Southeast and East Asian region, was observed. The worldwide propagation of waves retards the atmosphere and ionosphere. The EPBs created intense L-band magnitude scintillations at the middle-to-low latitudes. The signal fading depth was found to be ~ 16 dB. The conclusion of the research indicates that the eruption would indirectly affect the satellite communication links in areas greater than ten thousand kilometers away [18]. Themens et. al., (2022) tracked the travelling ionospheric disturbances (TIDs) related to the Tonga Hunga-Hunga Haapai submarine eruption. For this purpose, globally over 4,735 distributed GNSS receivers were used to extract measurements. Two main findings were concluded in this paper. Two varying Large Scale travelling ionospheric disturbances (LSITDs) and multiple Medium Scale travelling ionospheric disturbances (MSITDs), which travel radially outward from the eruption site, are identified. The LSITDs of wavelength >1,600 km are observed travelling at speeds of ~950 and ~555 m/s. Meanwhile, the MSTIDs with speeds ranging between 200 and 400 m/s are observed 6 hrs after the eruption [19].

Li et. al., (2023) made use of the data sets of the BeiDou Navigation Satellite System to validate the total electron content (TEC) variation over the regions of China. The most reliable need for choosing this particular data set over the other ground-based TEC data is the advantage of providing more precise calculations of the travelling speed of the TIDs. It also facilitates the monitoring of TEC variations for fixed ionospheric piercing points. The atmospheric pressure records were examined in which the Lamb waves crossed by the same station four times with a constant speed of 310 m/s. But upon TEC result comparison, the disturbances pass over China with four different speeds. Two among them were short- path direction and long-path direction [20]. Lin et. al., (2013) focused on analyzing the ionospheric TEC data of a volcanic eruption in Colima, Mexico. The data between 05 to 10 January 2013 were used for analysis by using two-dimensional principal component analysis (2DPCA) and revealed a TEC anomaly. The anomaly was identified from 21:00–21:05 (UTC) on 06 January 2013 over the volcano. It lasted for about five minutes, during which the Equatorial ionization anomaly (EIA) could be observed. The major reason for its occurrence could be the shock acoustic waves due to induced seismic activities. The study proved the possibility of monitoring and predicting the eruptions and their related seismic activities [21].

Panda et. al., (2015) focused on establishing ionospheric models over the low latitudes. This area is concerned as the low latitude regions and its related characteristics vary from that of other parts the globe. This is due to the intrusion of equatorial electrodynamics. Furthermore, it is hard to define a low-latitude representative ionosphere map. The parameters varying day to day and their ease of susceptibility to any solar-terrestrial phenomena play a major role in the above-mentioned difficulty. The study indicates the research on the contribution of the various institutions and organizations in India in theoretical, experimental and modelling tasks on upper atmosphere and ionospheric features [22]. Fuso et. al., (2024) made use of the existing Variometric Approach for Real-Time Ionosphere Observation (VARION) algorithm for the prediction of TEC variations. The 2015 Illapel earthquake and tsunami analysis were used as input features for the machine learning algorithm. The inputs were ascertained from 115 GNSS stations, as a high amount of data allows the exploration of VARON-based machine learning classification for TEC perturbation estimation. The given data has a high impact and strength. The VARON-generated observations were further given as input to models like Random Forest and XGBoost. In conclusion, the model could be integrated to act as a real-time early warning system due to its low computational time [23].

## 2. Data and methodology

In this research paper, we investigated four large volcanic eruptions that took place at various volcanic centers of the Indonesian Belt, one volcano in Italy and one more volcano in Caribbean, a seismically active region where great eruptions often produce powerful Atmospheric Gravity Waves (AGWs). AGWs rise upward and profoundly disturb the ionosphere, creating EPBs —plasma-density-depleted areas that have a great impact on satellite communication and navigation signals. To improve the understanding and forecasting of these disturbances, we examined the influence of volcanic eruptions on TEC changes and stressed the significance of sophisticated predictive modelling in predicting ionospheric activity under natural hazard events.

We acquired TEC data using IONOLAB, a dedicated program created by the IONOLAB team at Hacettepe University, Turkey. IONOLAB applies dual-frequency GNSS observations to calculate vertical TEC with fine temporal resolution and high accuracy. TEC measurements were obtained mainly from the BAKO GNSS station (6.4875° S, 106.8496° E) for the Mt. Kelud, Mt. Sinabung, Mt. Semeru and Mt. Ruang volcanos, other locations such as Mount Etna volcano (37.7518° N, 14.9947° E) located in Italy and Mount La Soufriere located on the island of Saint Vincent in the Caribbean (13.333° N, 61.183° W) TEC values were taken from Global Ionospheric Map (GIM). The daily dataset for each day amounted to 576 observations taken at 2.5-minute intervals, allowing for high-frequency monitoring of short-lived eruption-induced TEC variations.

Integration of heterogeneous data sets was achieved via a common hourly temporal resolution during pre-processing. Hourly TEC measurements were obtained using the pre-filtered and averaged data from the initial 2.5-minute time series. Data on seismic events included several observations per hour (earthquake count, average magnitude, average depth, and seismic energy), since events could occur within one hour of observation. On the other hand, gas emissions data and thermal anomaly had an hourly time scale initially; therefore, no aggregation was needed.

An hourly pre-merged dataset was built to combine all input features at an hourly time scale before generating features and training the model. Interpolation of any kind was not necessary because the variables obtained after pre-processing shared a common time resolution. This method was chosen to avoid artificial variations in the data over time while maintaining temporal consistency in time scale.

Additionally, an hourly time scale was used because eruption-related parameters were available hourly, and the goal of this study is to investigate the evolution of TEC variation on hourly and daily time scales. For predictive modelling, we utilized individual machine learning methods and a Hybrid structure:

### Individual models

**LightGBM** (Light Gradient Boosting Machine): A tree-based ensemble learning technique optimized for accuracy and efficiency. It is well-suited to deal with non-linear correlations between TEC and external drivers, and offers feature importance insights for eruption parameters [24].

**LSTM** (Long Short-Term Memory networks): A neural network architecture specifically intended for sequential data. LSTM detects the temporal dependencies and long-term memory in TEC changes, which are important during abrupt post-eruption instability [25].

### Hybrid model (LightGBM–LSTM)

Here, the weighted average ensemble framework was employed by integrating the strengths of LightGBM and LSTM. Instead of the sequential Hybridization method, this method combines both models results using optimal determined weight of the model. Its advantages involve improved generalization, as it reduces the overfitting to the specific data pattern with improved stability.

The models were trained using three months of pre-eruption TEC collected from the IONOLAB website (https://www.ionolab.org/) & GIM website (https://cdaweb.gsfc.nasa.gov/pub/data/gps/tec15min_igs/) and other eruption parameters. TEC is the measure of electron density in the ionosphere. It is relevant for this task because volcanic eruptions can affect the ionosphere through disturbances caused by magma movements and gases released during the process. Seismic parameters, such as number, depth, and average magnitude of earthquakes collected from the IRIS website (https://ds.iris.edu/), give valuable information about what happens underground since any changes in all of these indicators can be signs of pressure building up and magma moving inside the volcano. Finally, there are gas emission parameters collected from the Copernicus Atmosphere Monitoring Service (https://ads.atmosphere.copernicus.), namely, CO_2_ and SO_2_. An increase in their amounts is a sign of magma degassing and moving upwards as it approaches the surface of the volcano. Additionally, thermal emission parameters from NASA FIRMS(https://firms.modaps.eosdis.nasa.gov/) can help us find out whether magma is approaching the surface based on an increase in surface temperature.

Model performance was assessed against datasets for 6–7 days subsequent to every volcanic eruption, when ionospheric disturbances are most salient.

**Fig 1:**
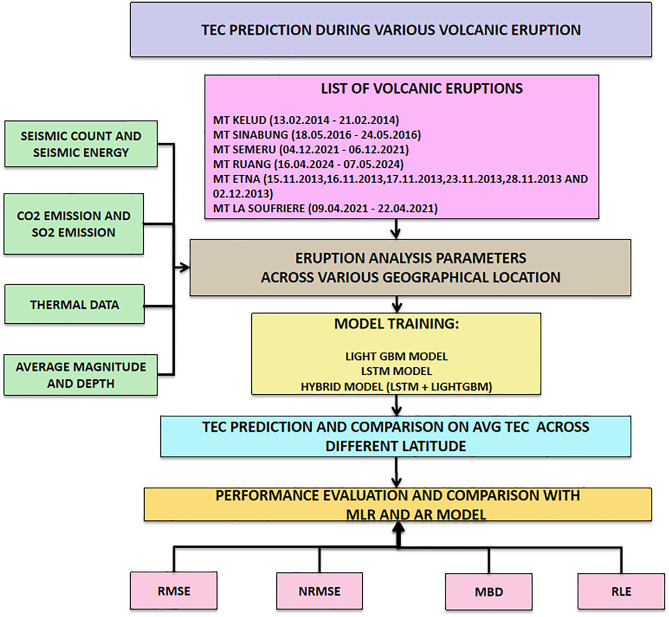
TEC Prediction during Volcanic Eruptions in the world across different years.

The individual model analysis indicated that LightGBM was good at picking up short-term fluctuations and making fast predictions, whereas LSTM was efficient in encoding long-range temporal dependencies and post-eruption recovery stages. The Hybrid (LightGBM–LSTM) ML model, nevertheless, outperformed both single models consistently, yielding lower values of RMSE, NRMSE, MBD and RLE indicative of its superior ability to emulate the non-linear, localized, and dynamic ionospheric responses caused by volcanic eruptions. Here, Fig 1 represents the TEC Prediction during Volcanic Eruptions in the world across different years.

### 2.1. LightGBM

LightGBM is a versatile supervised learning algorithm that can be utilized for both classification and regression problems. The fundamental concept behind LightGBM is to construct a group of decision trees using gradient boosting, which improves computational efficiency and accuracy. It predicts continuous technology values and also employs regularization techniques for broadening its applicability. The purpose of LightGBM, as shown in Equation 1, is to minimize the difference between predicted and actual values, while also considering the complexity of the model. The boosting process improves the model’s capacity to generalize well to different variations of technology. It explains the loss function employed in LightGBM, which is advantageous in handling the variations that occur during volcanic eruptions.

Equation 2 determines the gain required at the leaf-wise growth strategy, which determines the split node. To resolve the Optimization challenge, LightGBM employs L1 and L2 regularization methods, which are depicted in Equation 3 using Lagrangian multipliers.

For non-linear relationships, LightGBM can utilize feature transformations and custom loss functions to enhance predictive accuracy. An important hyperparameter ‘num_leaves’, influences the complexity of the tree, a balance between precision and overfitting. LightGBM employs histogram-based learning, preserving the accuracy. Table 2 shows the list of hyperparameters used for performing TEC prediction using the LightGBM model.

**Table 2:** Hypertuned Parameters used to train the LightGBM Model.

Parameter	Value	Parameters	Values
Objective	Regression	Learning Rate	0.01–0.03
Metric	RMSE	Maximum Depth	5–15
Boosting Type	GBT	Minimum Child Sample	10–20
Number of Leaves	31–127	Alpha,Lambda	0–0.3
N_estimators	150–210	Max_depth	3–6

LightGBM is commonly employed for real-time predictions because of its efficiency and flexibility. While predicting TEC variations during eruptions, it makes use of TEC patterns to provide dependable forecasts, enhancing the robustness of satellite-based systems. LightGBM employs various formulas as mentioned below:

### Objectives of LightGBM

#### Regression objective.

It is used for predicting the continuous values of data rather than the categorical variable. It minimizes the difference between the predicted value and actual value by optimizing the loss function, like Root Mean Square Error (RMSE).


$$\begin{array}{c} L(\theta )=\sqrt{\frac{\sum (y_{i}{-{\hat{y}}_{\text{i})}}^{\text{2}}}{N}} \end{array}$$ (1)

Where,

L(θ) represents loss function,

θ represents model parameters,

N is the total number of samples,

yᵢ is the true value,

ŷᵢ is the predicted value.

#### Growth strategy.

LightGBM expands trees in a leaf-wise manner, it reduces overfitting by splitting the node with the highest gain.


$$\begin{array}{c} \text{Gain }=\frac{\text{1}}{\text{2}}*[(\frac{\text{G}\text{l}\text{2}}{\text{H}\text{l}})+(\frac{\text{Gl2}}{\text{Hl}})-(\frac{(\text{G}\text{l}+\text{Gl})\text{ 2}}{\left(\text{Hr}+\text{Hr}\right)})]-\gamma \end{array}$$ (2)

Where,

G_l_ and Gr represents the sum of gradients in the left and right node,

H_l_ and H_r_ is the sum of second order derivatives of left and right node,

γ is the Regularization parameter.

#### Regularization in LightGBM.

Regularization is method used to avoid Overfitting. L1 regularization (Lasso) is used for feature selection.


$$\begin{array}{c} \text{L }=\Sigma (\text{yi}-\hat{y}\text{i}{)}^{\text{2}}+\lambda \Sigma |\text{wj}| \end{array}$$ (3)

Where,

L represents the total loss function,

(yᵢ – ŷᵢ)^2^ is the sum of squared errors,

w_j_ is the sum of absolute values.

**Fig 2:**
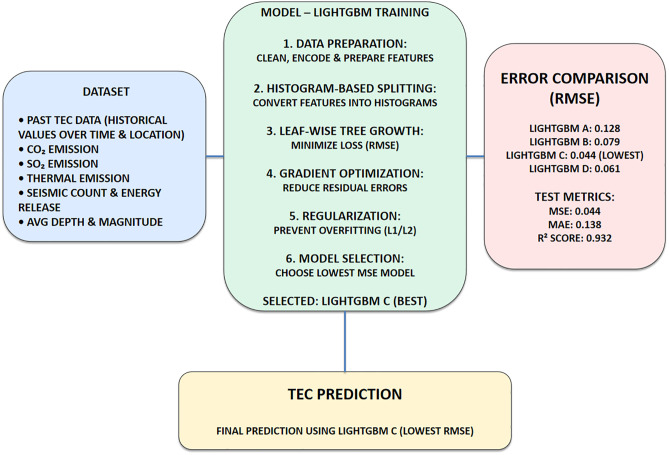
LightGBM (Light Gradient Boosting Machine) Overview.

LightGBM starts by loading the dataset, which is then pre-processed to handle missing values, encode categorical data, and normalize features if needed. Next, feature selection removes unnecessary attributes to improve efficiency. The model is initialized with parameters like learning rate and tree depth. Unlike traditional models, LightGBM grows tree leaf-wise, reducing errors faster. It also uses histogram-based binning to speed up training. During training, the model follows gradient boosting, where each new tree corrects mistakes from the previous ones. It adjusts weights to focus on difficult cases and continues until a stopping condition is met, such as reaching the iteration limit or detecting no further improvement. Once trained, the model is saved and used for predictions. Its performance is evaluated using metrics like accuracy, RMSE, or AUC, depending on the task. After validation, it is ready for real-world applications. The overview of LightGBM TEC prediction is illustrated in Fig 2.

### 2.2. LSTM model

This study leverages long short-term memory (LSTM), a distinct RNN, to estimate TEC values. LSTM is integrated with built-in memory that maintains information over extended time intervals, distinguishing the formation of equatorial plasma bubbles and other irregularities following a volcanic activity.

#### 2.2.1. Recurrent neural network architecture.

The established RNN data-processing sequence maintains a hidden state that serves as memory for all preceding elements. The computation at the respective time stamp t is:

#### 2.2.2. RNN hidden state update equation.

Equation 4 represents the RNN Hidden State Update,


$$\begin{array}{c} h_{t}=\phi \left(W_{hx}x_{t}+W_{hh}h_{t-\text{1}}+b_{h}\right) \end{array}$$ (4)

**Where**

- h_t_ is the hidden state at time step t,
- x_t_ is the input vector at time step t,
- h_t−1_ is the hidden state at the previous time step,
- W_hx_ is the input-to-hidden weight matrix,
- W_hh_ is the hidden-to-hidden recurrent weight matrix,
- b_h_ is the bias vector,
- ϕ(⋅) denotes the nonlinear activation function. In this study, the ReLU activation function is employed here.

#### 2.2.3. RNN output.

Equation 5 represents the RNN Output,


$$\begin{array}{c} y_{t}=w_{y\text{h}}{\text{h}}_{t}+b_{y} \end{array}$$ (5)

Where

$w_{y\text{h}}$ are the weight matrix,

$b_{y}$ are the bias vectors and

RNN encounter the vanishing gradient problem. In the course of training through backpropagation, the gradients are multiplied over several timestamps. If the gradients are small, they reduce exponentially, which makes it insignificant to modify the weights of the network. The LSTM architecture introduced by Hochreiter and Schmidhuber in 1997 was distinctly designed to overcome the problem of vanishing gradients.

### 2.3. LSTM architecture

The significant innovation in LSTM is its memory cell $c_{t}$ which sustains its state for an extended period. The inflow and outflow of information is coordinated by the three gates: Forget gate, Input gate and output gate [26]. The gates decide the matter of information which is to be retained, including or excluding.

#### 2.3.1. Forget gate.

Equation 6 represents the Forget Gate,


$$\begin{array}{c} f_{t}=\sigma (w_{f}.[{\text{h}}_{t-\text{1}},x_{t}]+b_{f}) \end{array}$$ (6)

Where $f_{t}$ denotes the forget gate at time step t. which decides how much of previous cell state to be retained or discarded. The σ represents the sigmoid activation function, producing values between 0 and 1. The term $[{\text{h}}_{t-\text{1}},x_{t}]$ is the concatenation of the previous hidden state and current input, while $w_{f}$ and $b_{f}$ represents the weight matrix and bias term associated with the forget gate.

#### 2.3.2. Input gate.

Equation 7 represents the Input Gate,


$$\begin{array}{c} i_{t}=\sigma (w_{i}.[{\text{h}}_{t-\text{1}},x_{t}]+b_{i}) \end{array}$$ (7)

Where $i_{t}$ denotes the input gate activation at time step t, which decides how much of the new information to attend to the cell. The σ represents the sigmoid activation function, producing values between 0 and 1, acting as a filter to control the flow of information. The term $[{\text{h}}_{t-\text{1}},x_{t}]$ is the concatenation of the previous hidden state and current input, while $w_{f}$ and $b_{f}$ represents the weight matrix and bias term associated with the input gate.

#### 2.3.3. Candidate cell state.

Equation 8 represents the Candidate cell state,


$$\begin{array}{c} {\tilde{c}}_{t}=tanh\left(W_{c}[h_{t-\text{1}},x_{t}]+b_{c}\right) \end{array}$$ (8)

Where ${\tilde{c}}_{t}$ denotes the cell state candidate at time step t, which represents a new, potential value to be added to the cell state. The tanh represents the hyperbolic tangent activation function, producing values between −1 and 1. The term$[{\text{h}}_{t-\text{1}},x_{t}]$ is the concatenation of the previous hidden state and current input, while $w_{c}$ and $b_{c}$ represent the weight matrix and bias term associated with the cell state candidate.

#### 2.3.4. Updating the cell state.

Equation 9 represents the updation of the cell state,


$$\begin{array}{c} c_{t}=f_{t}⊙c_{t-\text{1}}+i_{t}⊙{\tilde{c}}_{t} \end{array}$$ (9)

Where $c_{t}$ denotes the new cell state at time step t. This operation updates the memory of the LSTM by combining information from the previous cell state and the new candidate values. The $f_{t}$ is the forget gate, which decides how much of the previous cell state $c_{t-\text{1}}$ to retain. The $i_{t}$ is the input gate, which decides how much of the new candidate value ${\tilde{c}}_{t}$ to add to the cell state and the ⊙ is the Hadamard product.

#### 2.3.5. Output gate.

Equation 10 represents the Output Gate,


$$\begin{array}{c} o_{t}=\sigma \left(W_{o}[h_{t-\text{1}},x_{t}]+b_{o}\right) \end{array}$$ (10)

Where $o_{t}$ denotes the output gate activation at time step t, which regulates how much of the cell state contributes to the hidden state(output). The σ represents the sigmoid activation function, producing values between 0 and 1, acting as a filter to control the flow of information. The term $[{\text{h}}_{t-\text{1}},x_{t}]$ is the concatenation of the previous hidden state and current input, while $w_{o}$and $b_{o}$ represents the weight matrix and bias term associated with the output gate.

#### 2.3.6. Hidden state output.

Equation 11 represents the Hidden State Output,


$$\begin{array}{c} {\text{h}}_{t}=o_{t}⊙tan\text{h}(c_{t}) \end{array}$$ (11)

Where ${\text{h}}_{t}$ denotes the hidden state (or the output) of the LSTM at time step t. This value serves as both the output of the current cell and the hidden state passed to the next time step. The $o_{t}$ is the output gate, which decides how much of the cell state to reveal in the hidden state. The tanh function is applied to squish the cell state values between −1 and 1. Table 3 shows the values which have been given for training the LSTM model.

**Table 3:** Parameters used to train the LSTM Model.

Parameter	Value
Epoch	450–500
Batch	16
Optimizer	Adam
Loss Function	RMSE
Learning Rate	10^–4^–10^–2^
DropOut	0.1–0.5

### 2.4. LSTM model for TEC prediction

Precise estimation of ionospheric TEC is formulated as a supervised, multivariate time-series regression problem. The model is trained to predict tec from previous data. The target variable consistent TEC value at time t + 1.

#### 2.4.1. Input layer.

It contains 8 key measurements including past TEC, CO_2_ emission SO_2_ emission, thermal data, seismic count seismic energy, average depth and magnitude occurred during over the previous hours.

#### 2.4.2. First LSTM layer.

It explores the data hour by hour to identify initial pattern and relationship. The maintenance of the temporal sequence is essential for the sustenance of the legacy of states in subsequent layers.

#### 2.4.3. Second LSTM layer.

It performs the higher-level analysis for the first layer which integrates all the information into executive summary to make the necessary prediction

#### 2.4.4. Dropout layer.

During the training it aimlessly ignores the 20% interactions form the previous layer at each step so that it does not rely too intensively on executive path.

#### 2.4.5. Output layer.

Prediction of the real, continuous values of TEC for the next value of t + 1.

**Fig 3:**
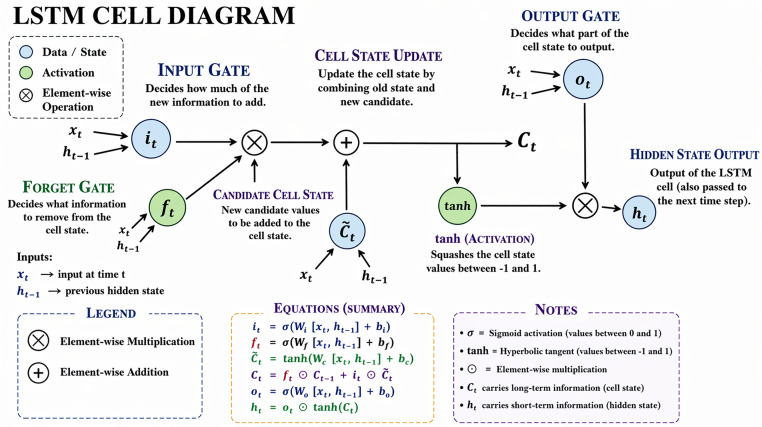
LSTM (Long Short-Term Memory network) overview.

The relationship between input parameters and tec is highly complex and non-linear data. The multigated LSTM model estimates these complex functions optimally. Its robustness to the time gap between eruption and their effect on TEC. LSTM yield the accurate and reliable predictions by overcoming vanishing gradients and longer-range temporal dependencies. The overview of LSTM TEC prediction is illustrated in Fig 3.

### 2.5. Hybrid model: (LSTM and LightGBM)

#### 2.5.1. Foundation for Hybrid model.

All the machine learning shows its strength in training for either classification or regression. LightGBM outperforms with structured and tabular data by its tree-based boosting system, but has a deficit with temporal dependencies. LSTM models are experts in sequence, but they are data-demanding and unstable with smaller and noisy data. The Hybrid model, with the collaboration of LightGBM and LSTM models, overcomes the limitations of each. LightGBM provides modeling of non-linear interactions among engineered features. LSTM contributes by modelling the temporal dynamics and memory of ionospheric variations by analysing the features.

#### 2.5.2. The Hybrid architecture.

LightGBM and LSTM are trained in parallel with the same dataset. LightGBM receives feature-engineered data, and LSTM receives the sequenced version of a subset of data. The final prediction is based on the weighted average of the individual models. Equation 12 represents the calculation of the final prediction of the Hybrid ML-DL Model for TEC,


$$\begin{array}{c} y_{Hybrid}=(w_{lgb}*y_{lgb}+w_{lstm}*y_{lstm})/(w_{lgb}+w_{lstm}) \end{array}$$ (12)

Where $y_{Hybrid}$ is the final predicted output. $w_{lgb}$ and $w_{lstm}\;$ denotes the weight of the individual LightGBM and LSTM models. $y_{lgb}$ and $y_{lstm}$ is the predicted values of individual models.

Averaging cancels out the uncorrelated errors, improves stability, and minimizes the variance associated with individual model predictions, leading to smoother and more reliable results and mitigating the problem of overfitting by balancing short-term and long-term prediction capabilities to specific data patterns using Hybrid models.

A comprehensive problem like TEC forecasting using a Hybrid approach is more imperative. This approach has superior predictive performance and robustness. It provides both the computational twin of the ionospheric system with key features and temporal evolutions leading to more reliable predictions during events like volcanic eruptions.

#### 2.5.3. Feature importance analysis.

In order to shed light on the significance of the different input variables for predicting the TEC, a feature-importance analysis was done based on the LightGBM component of the proposed Hybrid ML-DL framework. The LightGBM algorithm enables computing interpretable feature importance measures and, thus, provides a way of assessing the contribution of historical TEC values, atmospheric emissions, thermal anomalies, and earthquake parameters to the prediction process. Moreover, SHAP (SHapley Additive exPlanations) analysis has been applied to obtain an additional insight into the predictive model.

The suggested Hybrid ML-DL framework integrates LightGBM and LSTM components in parallel. While the two models use the same training set, the information learned is different: LightGBM relies on engineered tabular features, which include not only the TEC values but also geophysical variables, while LSTM learns directly from the time-series data. The predicted TEC value is produced based on the weighted average of the output of both models in accordance with Equation (12).

**Fig 4:**
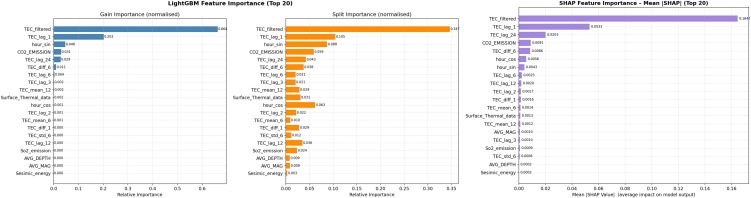
Normalized scores of Gain and Split importances derived from the LightGBM model and SHAP analysis.

**2.5.3.1. LightGBM feature importance:** Fig 4 presents the normalized scores of Gain and Split importance derived from the LightGBM model. Gain importance measures the importance of a variable based on its ability to reduce the prediction error, whereas Split importance measures the number of times a particular feature is selected in the trees.

From all the variables, the TEC_filtered turns out to be the most important variable and contributes around 66.4% of the total gain importance. Following TEC_filtered, there are TEC_lag_1 and TEC_lag_24. The dominance of these variables suggests that the recent history of TEC is the most important predictor of future TEC.

There are also some non-TEC variables that contribute to the model. The most important external feature is CO_2__EMISSION, followed by TEC_diff_6, hour_sin, and hour_cos. It can be concluded that atmospheric conditions and daily ionospheric variations add extra information. There are several volcanic and seismic-related features: SO_2__emission, Surface_Thermal_data, Seismic_energy, AVG_MAG, and AVG_DEPTH. The importance of these variables is comparatively smaller than the previous ones, but still they contribute to the prediction process.

**2.5.3.2. SHAP-Based Interpretation:** In order to further explore the effect of each feature, a SHAP analysis was conducted, and its outcomes are shown in Fig 4. The SHAP rankings largely coincide with those obtained for feature importance using LightGBM.

The highest SHAP values were observed for TEC_filtered, followed by TEC_lag_1 and TEC_lag_24, thus validating the fact that the most influential features in the prediction are the past TEC observations. From the set of external features, CO_2__EMISSION was found to have the biggest impact, followed by hour_cos, hour_sin, Surface_Thermal_data and SO_2From among the set of external variable__emission.

The high coincidence of the rankings from the two different methods provides more confidence about the robustness of the identified ranking of the features, and also proves that the past TEC plays the leading role in prediction.

**2.5.3.3. Reason for historical TEC features dominate:** An important result of both models is that historical TEC variables, especially TEC_filtered, TEC_lag_1, and TEC_lag_24, dominate the analysis. This behavior is anticipated since TEC is a geophysical parameter which depends on time.

The ionosphere does not undergo any sudden changes in general; the state of the ionosphere changes smoothly because of the impact of solar radiation, geomagnetic activity, atmospheric motion, and transport processes in the ionosphere. In other words, the current value of TEC carries a lot of information about TEC in the future. For this reason, the most recent TEC values are always among the best predictors.

The importance of TEC_lag_24 is significant because of the diurnal variability of the ionosphere, which is a known process. The TEC values are similar from day to day because of the diurnal variation of solar ionization. At the same time, the importance of temporal variables hour_sin and hour_cos highlights the importance of the daily periodical variations.

From the machine learning perspective, the historical TEC variables are autoregressive predictors which are directly connected with the target variable. This feature provides them with greater decreases in the prediction error in comparison with external variables; hence, these variables get higher importance.

**2.5.3.4. Contribution of volcanic and earthquake-related variables:** While the predictive power of historical observations of TEC is the greatest, the analysis shows that there is some complementary information provided by volcanic and seismic parameters.

From among the set of external variables, CO_2__EMISSION has the largest importance score, implying that changes in the atmosphere caused by volcanic eruption might somehow be related to the state of the ionosphere. Similarly, SO_2__emission and Surface Thermal data give additional information about volcanic processes happening at the Earth’s surface.

On the contrary, the variables related to earthquakes (Seismic energy, AVG_MAG, and AVG_DEPTH) have rather low importance scores. Nonetheless, this doesn’t mean that these parameters have little effect on TEC. As opposed to TEC, seismic and volcanic processes affecting the ionosphere are typically episodic. The influence of such parameters may thus appear smaller when analyzing the whole dataset. Still, the use of these variables can improve the precision of predictions by supplying additional information during periods of high geophysical activity.

Thus, these results show that volcanic and seismic parameters cannot substitute the predictive power of historical TEC observations but rather complement them by enabling the model to catch possible disturbances.

**2.5.3.5. Implications for the Hybrid ML–DL framework:** Feature importance analysis also helps to interpret the proposed Hybrid ML–DL model. The LightGBM part selects the best predicting variables and offers an interpretation of their contributions to TEC forecast predictions. As one can see, the historical values of TEC itself have the highest importance, but the other types of variables provide valuable supplementary data.

In spite of the fact that the calculation of feature importance applies only to the LightGBM model, the conclusions obtained could be applied to the whole Hybrid model since the LSTM part was also trained on the same underlying observations. LightGBM tries to find the best predicting variables, while the LSTM part learns about the temporal relations and complex non-linear dependencies existing in the data, which are not considered as lag features.

As a result, we could say that the enhanced quality of the predictions produced by the proposed Hybrid model is mainly explained by the high persistence of the TEC data, but volcano, atmosphere, and earthquake-related variables help to identify deviations from the normal ionosphere state.

### 2.6. Autoregressive (AR) model

#### 2.6.1. Foundation of AR model.

The Autoregressive (AR) model [27] is a conventional statistical time series technique used widely for modelling linear and temporal dependencies in sequential data [28]. In the context of ionospheric analysis, the AR model provides a simple yet effective way to capture short-term TEC variations, especially when the geophysical environment remains stable.

The ionosphere exhibits continuous temporal behavior, where the present TEC values are influenced by past values. The AR model captures this relationship and models the TEC variations as a linear combination of its previous values. Although volcanic eruptions generate significant non-linear perturbations through atmospheric gravity waves (AGW) and geomagnetic processes, the AR model provides a reference model to evaluate the contribution of the linear temporal relationships in TEC variations.

#### 2.6.2. Mathematical representation.

An AR model of order P, represented by AR(P) is given as:


$$\begin{array}{c} {\;X}_{t}\;=c+\sum i=\text{1}p\phi_{i}X_{t-i}+\in_{t} \end{array}$$ (13)

Where:

- X_t_ represents TEC at time t
- C is a constant term
- $p\text{h}i_{i}$ are autoregressive coefficients
- $epsilon_{t}$ is a white noise error term

This mathematical Equation 13 indicates that the present TEC value is dependent on a weighted sum of its previous values along with a stochastic error component.

#### 2.6.3. Model implementation strategy.

In this study, the AR model is implemented using AutoReg function from the statsmodels framework, maintaining consistency with standard statistical methodologies. The input to the model consists of past TEC values derived from the IONOLAB dataset. Unlike LightGBM and LSTM models, which incorporate external volcano-related parameters, the AR model relies on a univariate time series and focuses only on intrinsic temporal dynamics.

The preprocessing steps include:

- Noise reduction and smoothing of the TEC data.
- Downsampling from a 2.5-minute resolution to hourly intervals.
- Normalization for stable parameter estimation.

The model is trained using three months of pre-eruption TEC data to effectively capture the baseline ionospheric behavior under quiet conditions.

A variable lag selection strategy is utilized, with the autoregressive order p is chosen between 2 and 24. The upper bound is set to one diurnal cycle, allowing the model to capture daily periodic variations in TEC while avoiding overfitting and excessive model complexity.

#### 2.6.4. Prediction framework.

After training, the AR model is employed to forecast TEC values during the post-eruption period (6–7 days). Predictions are generated in a recursive manner, with each value based on previously observed or estimated TEC values.

This iterative approach enhances computational efficiency and is well-suited for short-term applications. However, due to its dependency on historical TEC data, the model does not incorporate external input parameters such as CO2, SO2 emission, thermal data, seismic count & energy and finally the average magnitude and depth for the prediction of TEC.

#### 2.6.5. Parameter configuration.

Table 4 represents the parameters used for the AR model:

**Table 4:** Parameters used for AR model.

Parameter	Value	Description
Model Type	AR(p)	Autoregressive model
Library	statsmodels	Statistical modeling package
Input Data	TEC	Univariate time series
Lag Order (p)	2-24	Number of past observations
Maximum Lag	24	Represents diurnal cycle
Training Data	3 months	Pre-eruption TEC
Prediction Horizon	6-7 days	Post-eruption period
Estimation Method	OLS	Least Squares optimization
Evaluation Metric	RMSE	Accuracy assessment

#### 2.6.6. Performance evaluation.

The performance of the AR model is evaluated using RMSE, along with other statistical metrics such as NRMSE, MBD, and RLE to ensure consistency with other models.

The AR model performs reasonably well under undisturbed ionospheric conditions, effectively capturing short-term temporal variations and regular daily patterns in TEC. However, its predictive accuracy decreases significantly during volcanic eruption periods.

The limitation is mainly due to:

- Inability to capture non-linear variations
- Lack of external inputs such as volcano parameters
- Dependency only on past TEC values

As a result, the AR model often underestimates sudden increases in TEC caused by eruption-related disturbances.

#### 2.6.7. Comparative role in the study.

In this study, the AR model is used as a reference model for comparison. It provides a benchmark for effectively evaluating the performance of advanced machine learning and deep learning models such as LightGBM and LSTM.

Although the AR model efficiently captures linear temporal dependencies, it is limited in its ability to represent:

- Complex non-linear ionospheric variations
- Long-term temporal dependencies
- External influence factors

As a result, its performance is inferior compared to both individual models and Hybrid (LightGBM-LSTM) framework, especially during ionospheric conditions caused by volcanic eruptions.

### 2.7. Multiple linear regression (MLR) model

#### 2.7.1. Foundation of MLR model.

Multiple Linear Regression (MLR) is a supervised statistical method which is widely used to model the relationship between a dependent variable and several number of independent variables [29–31]. MLR provides a simple yet effective framework to understand how TEC is influenced by several factors such as volcanic activity, environmental conditions, temporal factors and historical TEC values. Unlike AutoRegressive (AR) model which is based on univariate time-series approach, MLR considers and incorporates effects of multiple factors simultaneously more detailed representation of ionospheric variations.

The ionosphere is influenced by several natural phenomena such as volcanic eruptions, earthquakes, solar activity, atmospheric gravity waves and geomagnetic disturbances. Volcanic eruptions generate acoustic and gravity waves that are capable of depositing large quantities of ash, aerosols and gases in the atmosphere and disturb the ionosphere propagating upward. Such disturbances most of the time provide variations of the TEC. The MLR model incorporates volcanic indicators along with temporal and historical TEC information to provide an easily understandable framework to quantify the relative contribution of different factors affecting TEC before, during, and after volcanic eruptions.

The ionospheric responses to volcanic eruptions are highly nonlinear and complex. The MLR is an important baseline model due to its simplicity, transparency and ability to identify statistically significant relationships between TEC and the predictor variables. Thus, it can be used as a benchmark to assess the effectiveness of advanced machine learning and deep learning techniques.

#### 2.7.2. Mathematical representation.

The Multiple Linear Regression model is expressed as:


$$\begin{array}{c} {\;Y}_{t}=\beta_{\text{0}}+\beta_{\text{1}}X_{\text{1},t}+\beta_{\text{2}}X_{\text{2},t}+\ldots +\beta_{n}X_{n,t}+\varepsilon_{t} \end{array}$$ (14)

Where:

- $Y_{t}$ represents the TEC at time t
- $\beta_{\text{0}}$ is the intercept term
- $\beta_{\text{1}},\beta_{\text{2}},...,\beta_{n}$ are the regression coefficients corresponding to the predictor variables
- $X_{\text{1},t},X_{\text{2},t},\ldots ,X_{n,t}$ denote the predictor variables at time t
- $\epsilon_{t}$ represents the random error term at time t

The above equation models TEC as a linear combination of multiple input variables, where each regression coefficient quantifies the contribution of the corresponding predictor toward TEC variability.

#### 2.7.3. Ridge Regression regularization.

Ridge Regression is a generalization of the ordinary MLR used to make the model more stable and less sensitive to over fitting. Ridge Regression adds an L2 penalty term to the cost function:


$$\begin{array}{c} J(\beta )=\sum_{i=\text{1}}^{N}(y_{i}-{\hat{y}}_{i}{)}^{\text{2}}+\lambda \sum_{j=\text{1}}^{n}\beta_{j}^{\text{2}} \end{array}$$ (15)

Where:

- J(β) = objective/ cost function
- N = number of observations (samples)
- n = number of predictor variables
- $y_{i}$ = actual TEC value for the $i^{t\text{h}}$ observation
- ${\hat{y}}_{i}$ = predicted TEC value for the $i^{t\text{h}}$ observation
- $\beta_{j}$ = regression coefficient of the $j^{t\text{h}}$ predictor
- λ = regularization parameter controlling the penalty strength

The Regularization term corrects and simplifies large coefficients and improves the generalization of the model, especially when predictor variables are highly correlated.

#### 2.7.4. Model Implementation strategy.

In this work, the MLR model is implemented using the Ridge Regression algorithm available in the Scikit-learn machine learning framework. The model is provided with volcanic, time and ionospheric variables.

The predictor variables are:

- Seismic Count
- Mean Depth of Earthquake
- Average Magnitude of Earthquake
- Seismic Release Energy
- CO_2_ Emission
- SO2 Emission
- Surface Thermal Observations
- Features of Seasonal and Daily Cycles
- History of TEC Lag Variables
- TEC Rolling Average and Standard Deviation

The dataset is processed well before training to enhance the data quality and the performance of the model. We deal with missing values by interpolation and forward/backward fill. We have used sine and cosine transformations to create sequential cyclic features to hold the periodic behaviour corresponding to the daily and seasonal TEC variations. Historical TEC lag variables and rolling statistical features are also built to capture short-term ionospheric memory effects. Finally, all features are standardised with the StandardScaler method to ensure numerical consistency during model training.

The model is trained on about 3 months of pre-eruption observations, so that it learns the baseline relationship between TEC and the predictor variables under relatively stable ionospheric conditions.

#### 2.7.5. Prediction framework.

Then, the trained MLR model is used to predict TEC of the post-eruption period, which is usually six to seven days after a volcanic eruption. The model does not use recursive forecasting methods, but estimates the TEC values directly at each time stamp according to the available predictor variables.

The MLR model can also respond in a better way to disturbances associated with eruptions, compared to conventional autoregressive approaches, by incorporating volcanic activity indicators along with historical TEC information. However, since TEC is assumed to be linearly related to the predictor variables, the model may not fully capture complex ionospheric responses associated with atmospheric gravity waves, geomagnetic interactions and other non-linear processes triggered by volcanic eruptions.

To prevent information leakage, a chronological train-test split was performed. The MLR model was trained with pre-eruption observations only and tested on the time after the eruption. This setup simulates the practical forecasting scenario where the model has to learn the background TEC-volcanic relationship from historical data and then generalize to disturbed ionospheric conditions after the eruption.

#### 2.7.6. Parameter configuration.

Table 5 presents the parameters used for training and evaluating the MLR model.

**Table 5:** Parameters used for MLR Model.

Parameter	Value	Description
Model Type	Ridge Regression	Regularized MLR model
Library	Scikit-learn	Machine learning framework
Target Variable	TEC	Dependent variable
Input Features	28 Features	Volcanic, temporal and TEC-derived variables
Regularization	L2 (Ridge)	Reduces overfitting
Alpha	100	Regularization strength
Feature Scaling	StandardScaler	Standardization of inputs
Training Data	3 Months	Pre-eruption observations
Prediction Horizon	6–7 Days	Post-eruption period
Estimation Method	Ridge Optimization	Regularized least squares
Evaluation Metrics	RMSE, NRMSE, MBD, NMBD, RLE, R²	Performance assessment

#### 2.7.7. Performance evaluation.

The MLR model is evaluated using Root Mean Square Error (RMSE), Normalized Root Mean Square Error (NRMSE), Mean Bias Deviation (MBD), Normalized Mean Bias Deviation (NMBD), Relative Logarithmic Error (RLE) and coefficient of determination (R^2^).

Overall, the MLR model shows a better predictive performance than the traditional AR models because it includes or considers several variables related to the volcanic activity, environmental conditions and sequential ionospheric behaviour. Ridge regularization reduces the variance of coefficients and hence avoids overfitting, which makes predictions more stable.

The MLR model has advantages such as interpretability, the ability to use multiple influencing factors together, and better robustness through regularization. However, the model still assumes linear relationships between TEC and predictor variables, which reduces the ability to represent highly non-linear ionospheric disturbances generated during major volcanic eruptions.

#### 2.7.8. Ridge MLR over plain MLR.

In the TEC prediction problem today, some of the predictor variables are expected to be correlated with each other. For example, lagged TEC variables, rolling statistics, and cyclic temporal variables might have overlapping information, and volcanic indicators like seismic count, seismic energy, gas emissions, and thermal observations might also show interdependencies during eruption periods. The regression coefficients obtained from ordinary least squares MLR may be unstable in such cases. In the post-eruption prediction stage, ridge regression was then employed to stabilize the coefficient estimation, reduce variance, and improve generalization performance.

#### 2.7.9. Comparative role in the study.

The MLR model is an intermediate benchmark between traditional statistical approaches and more sophisticated machine learning models. MLR provides a more informative picture of TEC variability than the AR model by considering volcanic, environmental and temporal parameters rather than entirely depending on previous TEC observations.

However, MLR is less effective than advanced approaches, such as LightGBM, LSTM, and the Hybrid LightGBM-LSTM model, which can capture complex non-linear relationships and long-term temporal dependencies. Thus, the MLR model provides a significant reference framework for the evaluation of the advantages of the modern machine learning techniques in the forecast of TEC variations related to volcanic eruptions and ionospheric disturbances.

The MLR model provides a transparent baseline but assumes a roughly linear and additive relationship between TEC and the predictor variables. In fact, ionospheric disturbances associated with volcanic eruptions may show threshold effects, delayed responses, non-stationary behaviour and non-linear interactions between volcanic forcing, atmospheric wave propagation and background geophysical conditions. Hence, while MLR provides an interpretable benchmark, it may not fully capture the complexity of eruption-driven TEC anomalies.

## 3. Results and discussion

### 3.1. Performance evaluation metrics

The performance of LightGBM, LSTM, Hybrid ML-DL Model, MLR, and AR is evaluated using the results of the statistical parameters. Through this, the promptness and veracity are estimated. The progress will be estimated using the following criteria:

#### 3.1.1. Root mean square error (RMSE).

It is the index used to calculate the correctness of the model for the predicted observations. RMSE demonstrates the error in the model. It illustrates a higher value for the large errors as the error terms are squared and their values are averaged. The reliability of the model is evaluated using RMSE.

The equation of RMSE is:


$$\begin{array}{c} \text{RMSE}=\sqrt{\frac{\text{1}}{\text{N}}(\sum_{\text{i}=\text{1}}^{\text{N}}{({\text{TEC}}_{\text{observed}}-{\text{TEC}}_{\text{predicted}})}^{\text{2}})} \end{array}$$ (16)

#### 3.1.2. Normalized root mean square error (NRMSE).

It is a quantitative measure that scales the extent of the observed data. This facilitates the comparison of various models constructed using various scales. The effectiveness of the models is validated by the lower values of NRMSE.

The equation of NRMSE is:


$$\begin{array}{c} \text{NRMSE }=\frac{\;\sqrt{\frac{\text{1}}{\text{N}}(\sum_{\text{i}=\text{1}}^{\text{N}}{({TEC}_{observed}-{TEC}_{predicted})}^{\text{2}})}\;}{{TEC}_{max}-{TEC}_{min}} \end{array}$$ (17)

#### 3.1.3. Mean bias deviation (MBD).

It measures the average deviation. It indicates the orientation of the model, whether it overestimates or underestimates the observed values. This depicts the bias orientation associated with the model.

The equation of MBD is:


$$\begin{array}{c} \;MBD=\frac{\text{1}}{N}\sum_{i=\text{0}}^{N}{TEC}_{observed}-{TEC}_{predicted} \end{array}$$ (18)

#### 3.1.4. Relative length error (RLE).

Relative Length Error (RLE) is a measure of the accuracy of measurement compared with respect to the true value. It can be represented as the ratio of the absolute error, which is defined as the difference between the observed and true value, over with the true value. By relativizing the error in relation to the size of the measured quantity, RLE facilitates a true comparison of accuracy on different scales and data sets.


$$\begin{array}{c} \text{ RLE}=\frac{{\text{TEC}}_{\text{observed}}-{\text{TEC}}_{\text{predicted}}}{{\text{TEC}}_{\text{observed}}} \end{array}$$ (19)

### 3.2. Analysis of TEC variations on six volcanic eruptions

This work includes the analysis of predicting TEC during four volcanic eruptions across the Indonesian belt, one volcanic eruption in Italy and one volcanic eruption in the Caribbean Island, with individual and Hybrid ML-DL models compared with the AR Model. The outcomes emphasize with the model’s capability to predict eruptions based on the changes in TEC, as during an eruption, there will be a rise in EPBs, which will cause a variation in TEC in the ionosphere, thereby affirming its utility for global TEC forecasting and space weather applications.

#### 3.2.1. Prediction of TEC during volcanic eruption at Mt Kelud (13-02-2014 to 21-02-2014).

Mount Kelud is a stratovolcano located in Kediri, East Java, Indonesia. It has a summit elevation of about 1,731 meters (5,715 feet). It is known for its explosive eruptions. Mount Kelud is considered one of the most active volcanoes in Indonesia because of its large eruptions, often producing large ash plumes and volcanic lightning throughout its history. More than 300 eruptions have occurred since 1000 AD. It last erupted on February 13^th^, 2014, which is considered the most significant eruption in Indonesia’s history. The eruption occurred on February 13, 2014, at 22:50 local time (UTC + 7). It resulted in the destruction of a large lava dome formed due to an effusive eruption in 2007 and the ejection of boulders, stones and ashes. The eruption sent volcanic ashes covering an area of about 500 kilometers in radius, and the total ejection was estimated from 120,000,000–160,000,000 cubic meters, being a VEI 4 eruption. The eruption reached its peak intensity during the night of 13^th^ −14^th^ February when the powerful explosion generated volcanic lightning and massive ash clouds. The ash also reached the Western region of Java and other neighboring islands by February 14^th^ afternoon, where traces of volcanic eruption ashes were found in the surroundings. The eruption caused fatalities, and more than 76,000 people were evacuated. The ashfall from the eruption caused severe destruction, such as the collapse of houses due to the weight of the ashes. The eruption also resulted in the closure of several international airports across Indonesia due to thick ash deposits. It also caused significant damage to a variety of manufacturing and agricultural industries. The eruption gradually subsided by February 14^th^ afternoon, although minor emissions continued for a short period. The ash fallout following the eruption continued on February 15^th^. The Indonesian military used water cannons to clear the road and was later involved in reconstruction efforts in the areas surrounding Kelud. After February 20^th^, most of the businesses and attractions that were closed due to ashfall had reopened, although cleaning operations were still ongoing. The volcano’s alert status was downgraded on 21st February, and the exclusion zone was reduced from 10 to 15 kilometers.

We analyzed TEC variations during the volcanic eruption at Mount kelud from February 05^th^ to February 26^th^, 2014 which showed its peak at February 13^th^ 2014, focusing on the performance of the Hybrid ML-DL Model compared with LightGBM, LSTM, AR and MLR model, particularly at volcanic eruption from February 13th to February 21^st^ 2014, caused significant ionospheric disturbances, challenging the predictive accuracy of both models. This disaster occurred for ten days in Indonesia. The performance metrics, summarized in Table 6, reveal the superior accuracy of the Hybrid ML-DL Model across all parameters.

**Table 6:** Performance evaluation and comparison during volcanic eruption at Mt. Kelud (05.02.2014 to 26.02.2014).

DATES	RMSE (TECU)					NRMSE					MBD (TECU)					RLE				
	Light GBM	LSTM	Hybrid	AR	MLR	Light GBM	LSTM	Hybrid	AR	MLR	Light GBM	LSTM	Hybrid	AR	MLR	Light GBM	LSTM	Hybrid	AR	MLR
2/5/2014	2.742	3.990	2.443	8.599	4.755	0.036	0.053	0.032	0.113	0.095	0.297	1.561	0.079	1.946	1.277	0.038	0.101	0.033	0.147	0.100
2/6/2014	2.581	3.227	2.015	5.264	4.895	0.040	0.051	0.032	0.083	0.099	0.082	1.007	0.146	0.366	0.891	0.046	0.052	0.031	0.073	0.091
2/7/2014	2.406	2.764	1.662	5.253	4.224	0.037	0.042	0.025	0.080	0.083	0.105	1.189	0.528	1.852	1.941	0.049	0.061	0.033	0.089	0.087
2/8/2014	3.782	3.792	3.158	5.447	5.414	0.053	0.053	0.044	0.076	0.107	0.273	0.595	0.108	0.626	1.126	0.078	0.090	0.064	0.110	0.138
2/9/2014	3.164	3.781	2.735	9.163	4.519	0.048	0.057	0.041	0.139	0.098	0.076	1.203	0.193	4.295	0.971	0.060	0.102	0.053	0.195	0.123
2/10/2014	2.453	3.364	2.079	6.686	4.313	0.037	0.050	0.031	0.100	0.090	0.161	0.507	0.035	1.524	0.039	0.038	0.082	0.031	0.116	0.104
2/11/2014	3.160	3.666	2.749	6.637	3.351	0.039	0.045	0.034	0.082	0.071	0.750	0.591	0.749	0.065	0.309	0.099	0.121	0.086	0.197	0.120
2/12/2014	5.880	3.043	2.969	12.465	7.041	0.065	0.034	0.033	0.138	0.123	2.452	1.120	0.929	10.654	4.576	0.089	0.052	0.048	0.202	0.135
2/13/2014	3.612	2.508	2.018	6.422	4.938	0.044	0.030	0.024	0.078	0.092	0.503	0.214	0.016	1.423	0.966	0.077	0.060	0.044	0.143	0.149
2/14/2014	3.085	2.941	2.417	5.187	3.982	0.043	0.041	0.034	0.073	0.078	0.179	0.599	0.318	0.932	1.081	0.067	0.068	0.047	0.101	0.097
2/15/2014	2.064	3.269	1.686	6.568	4.111	0.031	0.049	0.025	0.098	0.088	0.105	1.915	0.385	3.079	1.938	0.033	0.075	0.028	0.104	0.085
2/16/2014	3.188	3.926	2.667	7.197	5.187	0.053	0.065	0.044	0.119	0.116	0.363	2.268	0.948	1.770	2.131	0.058	0.087	0.044	0.127	0.098
2/17/2014	3.547	4.333	3.172	11.828	5.223	0.046	0.056	0.041	0.152	0.103	0.089	0.821	0.138	5.429	1.332	0.069	0.114	0.061	0.261	0.169
2/18/2014	3.324	3.889	2.348	5.739	5.087	0.044	0.051	0.031	0.076	0.098	0.575	0.602	0.321	2.379	0.654	0.056	0.069	0.037	0.122	0.155
2/19/2014	6.000	5.987	3.781	14.520	7.460	0.060	0.059	0.038	0.144	0.145	1.234	1.472	0.401	0.128	1.376	0.171	0.284	0.148	0.450	0.237
2/20/2014	3.578	4.110	2.916	6.566	5.596	0.040	0.046	0.032	0.073	0.116	0.308	0.567	0.309	1.450	2.755	0.084	0.124	0.072	0.139	0.155
2/21/2014	3.706	3.893	3.290	11.500	6.275	0.060	0.063	0.053	0.185	0.143	0.120	1.641	0.604	4.128	3.484	0.073	0.079	0.053	0.184	0.112
2/22/2014	2.702	3.634	2.319	7.263	3.853	0.037	0.050	0.032	0.100	0.076	0.292	1.551	0.544	5.572	0.913	0.047	0.058	0.038	0.100	0.076
2/23/2014	3.900	4.577	3.355	11.704	6.990	0.046	0.054	0.039	0.138	0.122	0.868	2.445	1.517	7.720	2.185	0.080	0.085	0.065	0.134	0.152
2/24/2014	7.546	5.434	4.834	12.923	7.552	0.078	0.056	0.050	0.134	0.117	2.841	3.035	2.180	9.817	2.698	0.088	0.075	0.053	0.147	0.170
2/25/2014	4.587	5.223	3.870	11.865	6.208	0.058	0.066	0.049	0.151	0.104	0.322	1.942	0.996	2.848	0.836	0.072	0.091	0.064	0.162	0.107
2/26/2014	4.614	4.584	3.756	12.187	5.661	0.062	0.062	0.050	0.164	0.216	0.476	1.541	1.245	4.824	5.661	0.059	0.055	0.039	0.187	0.243
**Average values during volcanic eruption days**	3.567	3.873	2.699	8.392	5.318	0.047	0.051	0.036	0.111	0.109	0.386	1.122	0.382	2.302	1.746	0.076	0.107	0.059	0.181	0.140
**Average values during Non-volcanic eruption days**	3.809	3.929	2.919	8.881	5.290	0.049	0.052	0.038	0.115	0.108	0.692	1.407	0.711	4.009	1.802	0.065	0.079	0.049	0.143	0.126
**Total** **Values**	4.879	4.866	3.736	11.825	5.302	0.058	0.059	0.045	0.145	0.108	0.917	1.931	1.191	5.586	1.779	0.084	0.098	0.065	0.191	0.132

By averaging the performance metrics, during the volcanic eruption, we analyzed that the ionosphere experienced considerable TEC deviations, with the Hybrid ML-DL Model achieving an RMSE of 2.699 TECU, significantly lower than the LightGBM’s RMSE of 3.567 TECU, LSTM’s RMSE of 3.873 TECU, AR model’s RMSE of 8.392 TECU and MLR model’s RMSE of 5.318 TECU. The NRMSE for the Hybrid ML-DL Model was 0.036, compared to 0.047 for LightGBM, 0.051 for LSTM, 0.111 for AR and 0.109 for MLR model, further emphasizing the Hybrid ML-DL Model’s superior accuracy. The MBD values also favoured the Hybrid ML-DL Model, averaging 0.382 TECU against the LightGBM’s 0.386 TECU, LSTM’s 1.122 TECU, AR model’s 2.302 TECU and MLR model’s 1.746 TECU, which consistently underestimated TEC. The Hybrid ML-DL Model recorded an RLE of 0.059, significantly under-performing the LightGBM’s RLE of 0.076, LSTM ‘s RLE of 0.107, AR model’s RLE of 0.181 and MLR model’s RLE of 0.140. Overall, the Hybrid ML model consistently demonstrated better accuracy across all metrics during this volcanic eruption compared to LightGBM, LSTM, AR and MLR.

**Fig 5:**
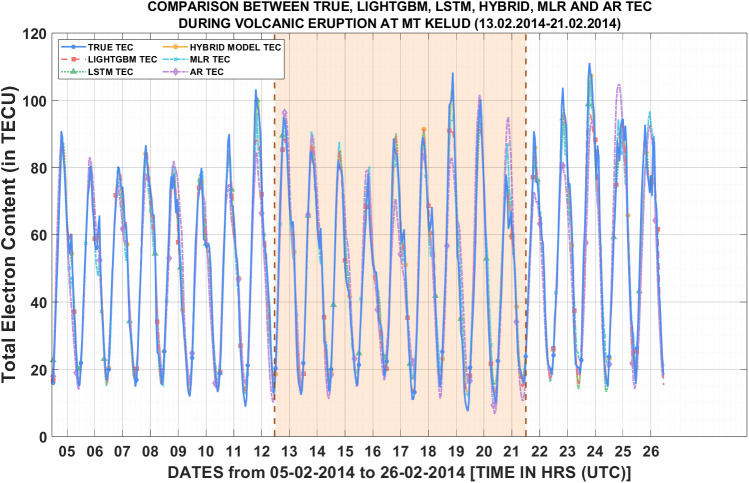
Comparison between TRUE, LightGBM, LSTM, Hybrid ML-DL Model, AR and MLR TEC during volcanic eruption at Mt. Kelud (13.02.2014 to 21.02.2014).

**Fig 6:**
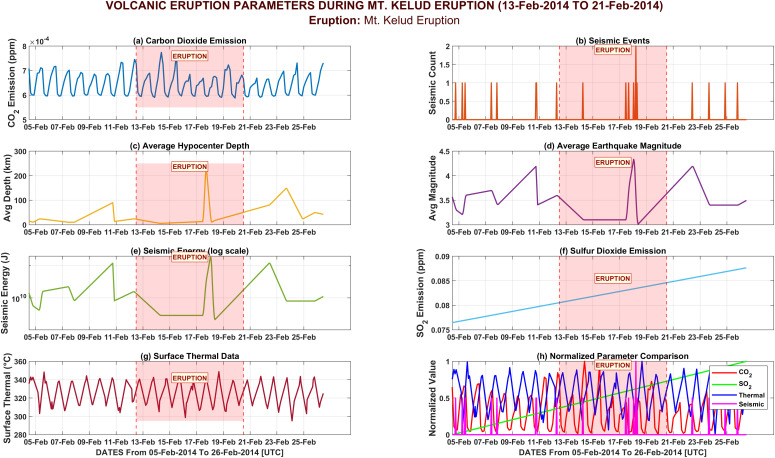
Parameters used during the volcanic eruption at Mt. Kelud (13.02.2014 to 21.02.2014).

Fig 5. illustrates TEC variations from February 05th to February 26^th^, 2014, and provides valuable insights into the ionospheric response to the volcanic eruption and the models’ performances. The TEC and Hybrid ML-DL Model Predicted TEC curves show a close match, accurately capturing daily variations. On February 19^th^, 2014, during the volcanic eruption, around 09:00 (UTC), a peak TEC of 108 TECU was observed due to the flare’s intensity. The Hybrid ML-DL Model closely predicted this at 102 TECU, whereas the LightGBM model with 100 TECU, the LSTM model around 91 TECU, the AR model underestimated it at 82 TECU and the MLR model predicted it around 94 TECU, which is due to the increase in seismic activity from 17^th^ February to 19^th^ February 2014, and there is a significant increase in CO2 and SO2 emissions. Here, the Hybrid ML-DL Model tends to predict the change in TECU correctly as compared with other individual models and also concludes that the AR model makes little worse to predict the changes as it follows a pattern in predicting TEC in all the days, unable to predict the change in TECU due to these parametric changes due to this volcanic eruption as they make predictions based only on TEC values. Fig 6 shows the Parameters during the volcanic eruption at Mt. Kelud.

Still, there’s a clear witness of a sudden increase in True TEC during the noon of 19^th^ February, but our Hybrid ML-DL Model could not predict the peak changes because of abrupt, unpredictable changes in eruption parameters. Even the AR and MLR models could not predict the peak caused in the True TEC values. Likewise, on February 19^th^ and February 24^th^ 2014, there had also been sudden events caused by variations in the parameters, which also could not be predicted by the Hybrid ML-DL Model and the other model.

**Fig 7:**
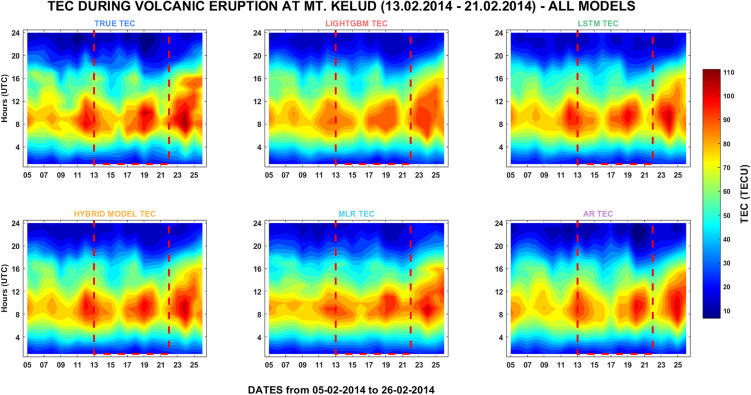
Comparison between TRUE, LightGBM, LSTM, Hybrid ML-DL Model, AR and MLR TEC during volcanic eruption at Mt. Kelud (13.02.2014 to 21.02.2014) using Contour plot.

Here, Fig 7 the contour plot of TRUE TEC, LightGBM, LSTM, Hybrid ML-DL, MLR and AR TEC during both normal and eruption days. Based on the maximum value and minimum value between TRUE TEC, Hybrid ML-DL Model, LightGBM, LSTM, MLR and AR TEC. Here, by analyzing the contour plot, we can determine the TECU as regions in the particular hours of the day, in the variation in colour (red for peak TECU, blue for low TECU).

During February 24^th^ 2014, the Hybrid ML-DL Model predicts the TECU values correctly except at the peak due to the parametric changes. On comparing with the TRUE TEC contour plot, the Hybrid ML-DL Model predicted TEC replicates it, as it matches almost all the regions with maximum accuracy. Here, the AR and MLR model don’t match with TRUE TEC, which indicates there is a slight difference between the TECU when comparing them in each hour (UTC)

**Fig 8:**
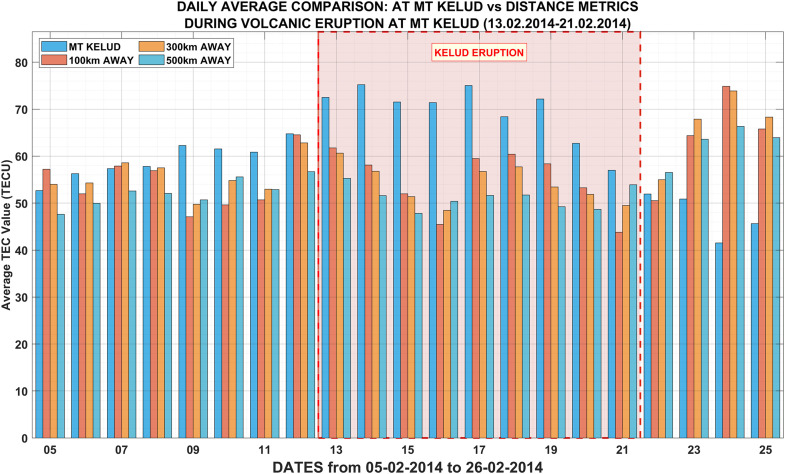
Comparison of daily average TEC during volcanic eruption at Mt. Kelud across various distances.

The Fig 8 above illustrates the daily average TEC values recorded at Mt. Kelud and the three distances away (100 km, 300 km, and 500 km) from it for the month of February 2014. The area marked by the shaded red region illustrates the duration of the volcanic eruption (13^th^–21^st^ February). It is observed that there are drastic changes in the TEC values for Mt. Kelud during the time interval, indicating disturbance of the ionosphere due to volcanic eruption. There are significant variations in the TEC values even at a distance of 100 km from the volcano. However, the changes become less pronounced with an increase in the distance from the volcanic source. Hence, at a distance of 300 km from the volcano, the changes in the TEC values become insignificant, while at 500 km distance, they remain constant. The above illustration clearly shows how the effect of a volcanic eruption decreases sharply with distance, thus causing minimum interference with the ionosphere farther from the volcano.

### 3.3. Prediction of TEC during volcanic eruption at Mt Sinabung (18-05-2016 to 24-05-2016)

Mount Sinabung is also known as Gunung Sinabung. It is an active stratovolcano located in the Karo plateau of North Sumatra, Indonesia. It is situated 40 kilometers away from the Lake Toba super volcano, being part of Indonesia’s volcanic arc, and it is almost 2,460 meters above sea level. The volcano has a complex structure with multiple volcanic craters. After being dormant for centuries, it reawakened in 2010, and continued eruptions occurred in 2010, 2013, 2014, 2016 and 2020. The eruptions have produced ash clouds that can reach heights of up to 10 kilometers, causing significant damage to the nearby settlements. The volcano’s activity has been continuously monitored by scientists and local authorities due to its proximity to the Indonesian Ring of Fire and its impacts on the surrounding regions. In 2016, Mount Sinabung produced one of its most destructive eruptions in recent years. The eruption sequence intensified in February 2016, with continuous dome growth and pyroclastic flows. On 21^st^ May 2016, the volcano exploded majorly around 16:48 local time (UTC + 7 hours). The eruption generated a huge pyroclastic flow which travelled up to 4.5 km down the southern slopes. From some studies, it has been estimated that approximately tens of millions of cubic meters of total erupted material have been emitted, making this a VEI 3 eruption. The eruptions rose several kilometers into the atmosphere, carrying large ash plumes and volcanic gases. This day marked the peak of the eruption, causing severe damage to the nearby settlements. By late May 2016, the eruption had gradually decreased, though smaller explosions and emissions continued. The impacts were devastating, it killed seven people and three were injured due to the overflow of pyroclastic flows within the designated danger zones. Thousands of people were evacuated to temporary shelters, and large agricultural lands were destroyed by hot gas clouds and thick ash deposits. Apart from the physical damage, the eruption caused a long-term economic hardship for the local population. Infrastructure like roofs of houses, public buildings, schools, roads and water systems collapsed under heavy ashfall, and the daily life of people in the surrounding communities was heavily disrupted. The alert status of the eruption was never downgraded in 2016; it remained in Level 4 until May 2019, when it was lowered to Level 3.

The TEC variations during the volcanic eruption at Mount Sinabung from May 18^th^ to May 24^th^, 2016, showed its peak on May 21^st^, 2016. This sudden variation caused significant disturbance to the ionosphere, challenging the predictive accuracy of the Hybrid ML-DL Model, LightGBM and LSTM model compared with the AR and MLR Model. The performance metrics, tabulated in Table 7, shows the predictive accuracy of the Hybrid ML-DL Model with other models during the volcanic eruptions at Mount Sinabung.

**Table 7:** Performance evaluation on Hybrid ML-DL model with other models during the volcanic eruption at Mt. Sinabung (16^th^ - 30^th^ May 2016).

DATES	RMSE (TECU)					NRMSE					MBD (TECU)					RLE				
	Light GBM	LSTM	Hybrid	AR	MLR	Light GBM	LSTM	Hybrid	AR	MLR	Light GBM	LSTM	Hybrid	AR	MLR	Light GBM	LSTM	Hybrid	AR	MLR
5/16/2016	2.169	2.250	1.626	3.407	2.700	0.042	0.044	0.032	0.067	0.129	0.367	0.900	0.132	1.819	0.924	0.368	0.444	0.367	0.623	1.897
5/17/2016	2.797	3.751	2.546	6.530	3.431	0.066	0.089	0.060	0.155	0.180	0.441	0.370	0.367	1.318	0.895	0.219	0.341	0.200	0.348	0.254
5/18/2016	1.750	2.050	1.488	5.545	2.980	0.033	0.039	0.028	0.105	0.161	0.615	0.464	0.415	0.504	0.958	0.448	0.698	0.432	1.409	0.433
5/19/2016	1.649	1.873	1.428	5.017	2.281	0.041	0.047	0.036	0.126	0.146	0.341	0.798	0.428	3.576	1.329	0.237	0.340	0.222	0.420	0.291
5/20/2016	1.938	1.964	1.177	4.995	2.637	0.039	0.040	0.024	0.100	0.137	0.508	0.977	0.010	1.601	0.388	0.110	0.255	0.070	0.325	0.213
5/21/2016	1.853	1.796	1.399	4.020	2.546	0.045	0.044	0.034	0.098	0.151	0.473	0.566	0.006	2.681	1.279	0.521	0.417	0.359	1.017	0.516
5/22/2016	1.988	2.102	1.686	4.276	2.155	0.042	0.044	0.036	0.090	0.112	0.447	0.105	0.058	1.273	0.552	4.638	5.667	4.617	8.918	1.021
5/23/2016	2.015	2.050	1.712	7.995	3.443	0.046	0.047	0.039	0.184	0.223	0.878	0.920	0.733	3.831	1.366	1.304	1.552	1.217	1.379	0.685
5/24/2016	1.786	1.653	1.495	2.815	2.399	0.045	0.041	0.037	0.070	0.159	0.779	0.689	0.561	2.139	1.081	0.829	0.667	0.613	1.351	0.582
5/25/2016	1.676	1.916	1.262	6.115	2.291	0.040	0.046	0.030	0.148	0.146	0.638	0.555	0.207	1.158	0.238	3.269	5.137	3.245	5.929	0.939
5/26/2016	1.797	2.542	1.456	2.492	2.451	0.043	0.060	0.034	0.059	0.151	0.185	0.518	0.206	0.549	0.028	0.103	0.205	0.076	0.256	0.274
5/27/2016	1.484	2.203	1.114	2.785	2.706	0.036	0.053	0.027	0.067	0.171	0.138	0.313	0.154	1.403	0.722	0.102	0.165	0.083	0.212	0.236
5/28/2016	2.001	3.282	1.868	8.336	3.548	0.049	0.080	0.046	0.204	0.205	0.505	0.252	0.491	0.625	0.489	0.089	0.173	0.079	0.299	0.198
5/29/2016	1.775	1.933	1.484	3.108	2.710	0.042	0.045	0.035	0.073	0.156	0.095	0.249	0.231	0.213	0.974	0.075	0.083	0.053	0.172	0.143
5/30/2016	2.162	2.120	1.854	3.638	2.617	0.051	0.050	0.044	0.086	0.330	0.160	0.132	0.106	0.359	2.617	0.157	0.254	0.136	0.613	0.285
**Average values during volcanic eruption days**	1.794	2.015	1.422	4.605	2.634	0.041	0.046	0.033	0.105	0.155	0.500	0.590	0.278	1.871	0.993	1.156	1.510	1.093	2.122	0.535
**Average values during Non-volcanic eruption days**	2.181	2.667	1.876	5.004	2.779	0.050	0.062	0.043	0.117	0.162	0.314	0.380	0.265	0.867	0.669	0.182	0.259	0.167	0.411	0.565
**Total** **Values**	1.735	2.152	1.466	4.205	2.678	0.043	0.054	0.036	0.105	0.171	0.290	0.237	0.267	0.672	0.912	0.745	1.038	0.698	1.395	0.461

By analyzing the average of performance metrics of the Hybrid ML-DL Model with the other models during the eruption period, we can see that the Hybrid ML-DL Model achieves an RMSE of 1.422 TECU, which is significantly lower than that of LightGBM with 1.794 TECU of RMSE, LSTM with 2.015 TECU of RMSE, AR Model with 4.605 TECU of RMSE and MLR Model with 2.634 TECU of RMSE. The NRMSE of the Hybrid ML-DL Model was 0.033, which further highlights the higher accuracy of the Hybrid model over LightGBM with an NRMSE of 0.041, LSTM with 0.046 NRMSE, AR Model with a NRMSE of 0.105 and MLR model with NRMSE of 0.155. Furthermore, the MBD of the Hybrid ML-DL Model was 0.278 TECU, which again demonstrates the superior accuracy of the model over LightGBM with 0.5 TECU of MBD, LSTM with a MBD of 0.59 TECU, AR with 1.871 TECU of MBD and MLR with 0.993 TECU of MBD. The LightGBM’s RLE was 1.156, LSTM’s was 1.51 of RLE, the AR model’s RLE was 2.122 and the MLR model’s RLE was 0.535, which is higher than that of the Hybrid ML-DL Model with a RLE of 1.093, emphasizing its better accuracy over other models. On the whole, analysis of the performance metrics illustrates the superior accuracy of the Hybrid ML-DL Model over other models in terms of all the performance metrics for the prediction of TEC variations during this volcanic eruption.

**Fig 9:**
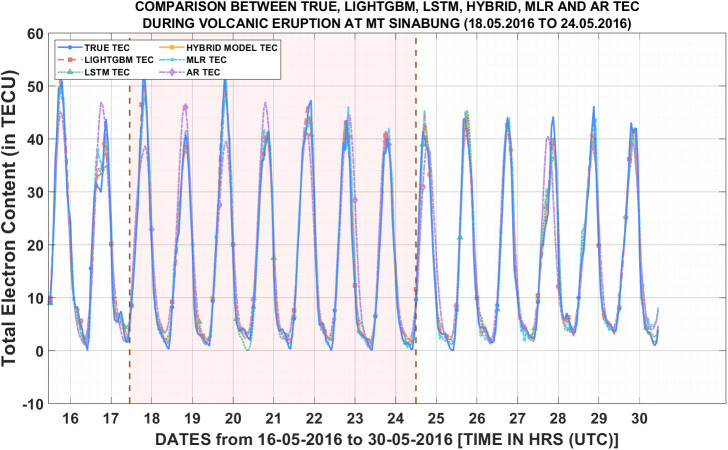
Comparison between True, Hybrid ML-DL Model, LightGBM, LSTM, MLR and AR TEC during the volcanic eruption at Mt Sinabung (16.05.2016-30.05.2016).

**Fig 10:**
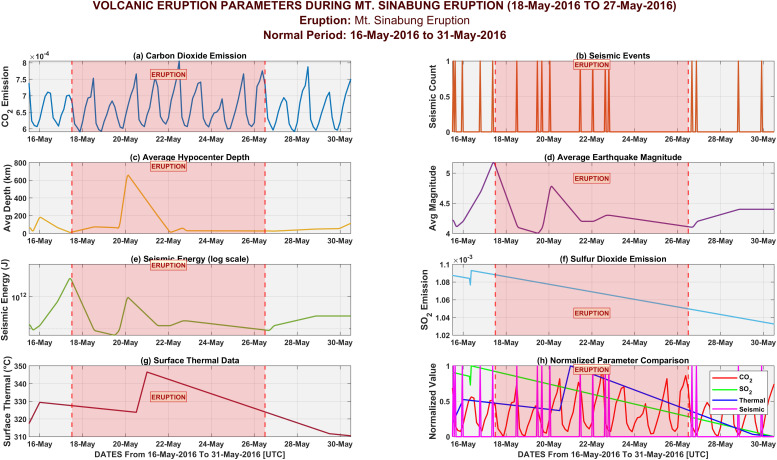
Eruption parameters which are analyzed during the volcanic eruption at Sinabung (18.05.2016 to 27.05.2016).

Fig 9 illustrates the TEC variations from May 16^th^ to May 30^th^, 2016, and provides significant knowledge about the ionosphere’s behavior and the performance of the Hybrid ML-DL Model. From the graph, we can see that the true TEC curve and the Hybrid ML-DL Model’s predicted TEC curve match closely, precisely capturing the daily variations of TEC. The effect of the volcanic eruption on the TEC variation showed its peak of 53.09 TECU on May 18^th^, 2016, around 7:00 (UTC). The Hybrid ML-DL Model predicted it as 51.502 TECU, which is very close to the true TEC value compared to the LightGBM’s predicted value of 50.33 TECU, LSTM’s predicted value of 47.26 TECU, the MLR model’s predicted value of 45.381 TECU and the AR model’s predicted value of 32.5 TECU. This highlights the superior accuracy of the Hybrid ML-DL Model over other models in predicting TEC variations in the ionosphere caused by gravity waves produced during the volcanic eruption. Fig 10 shows the eruption parameters which are analyzed during the volcanic eruption at Mt. Sinabung.

On May 22^nd^, 2016, there was an increase in CO2 emissions and the number of seismic events across the geographic location. The Hybrid ML-DL Model was able to predict the change closely, while the AR Model was not able to predict the sudden change in TEC, which are affected by this effect. Likewise, on May 20^th^, there was a sudden peak at 7:00 (UTC), and this was also closely predicted by the Hybrid ML-DL Model. So, for this volcanic eruption, the Hybrid model has performed well in all instances compared to other models.

**Fig 11:**
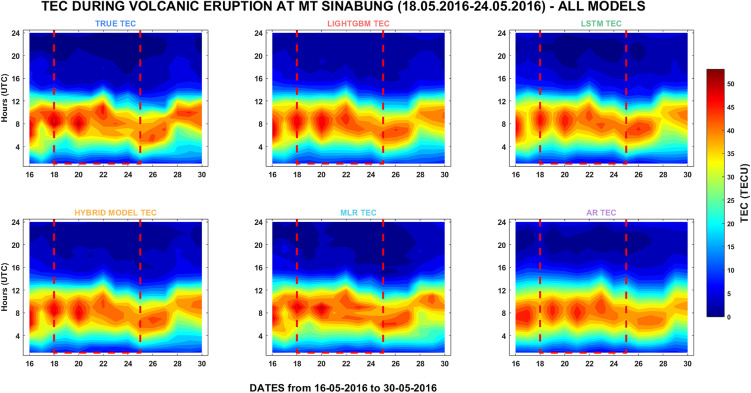
Comparison between True, Hybrid ML-DL Model, LightGBM, LSTM, MLR and AR TEC during volcanic eruption at Mt. Sinabung (18.05.2016 to 24.05.2016) as a contour plot.

Fig 11 represents the prediction of TEC variations during both normal and eruption dates as a contour plot based on the minimum and maximum value between the True TEC, LightGBM, LSTM, AR Model, MLR Model and the Hybrid ML-DL Model. By analyzing the contour plot, we can determine the TEC variations on an hourly basis by the variations in colour (Red for peak TECU, blue for low TECU).

On May 22^nd^, 2016, the Hybrid ML-DL Model predicts the TECU correctly except at the peak due to some parametric variations.

By comparing the True TEC plot with the Hybrid model’s TEC plot, we can observe that both plots closely match each other, illustrating the superior accuracy of the Hybrid model in capturing the hourly variations in TEC during the volcanic eruption. The AR model’s plot shows a vast difference from the True TEC plot, which indicates that the AR fails in capturing the hourly TEC variations precisely. The MLR plot slightly matches the True TEC plot compared to the AR model.

**Fig 12:**
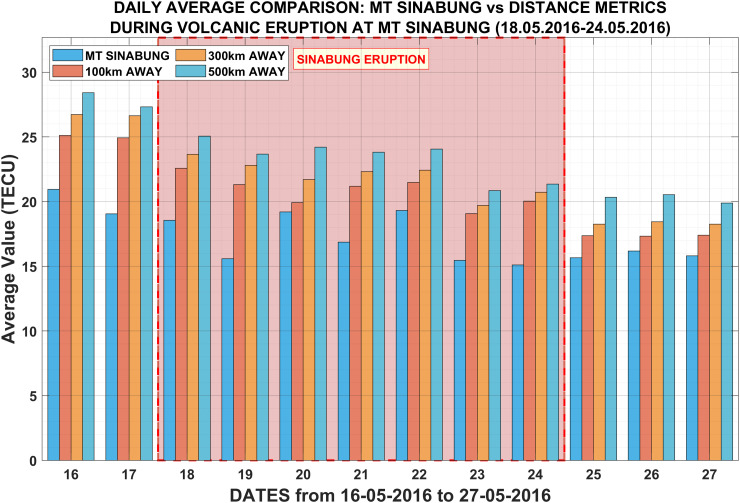
Comparison of daily average TEC during volcanic eruption at Mt. Sinabung across various distances.

Fig 12, shown above, presents the daily mean values of TEC obtained at Mt. Sinabung and the distances 100 km, 300 km, and 500 km away from it during the time interval from May 16^th^ to May 27^th^, 2016. The red-colored shaded portion indicates the time frame when the volcano is erupting (May 18–27, 2016). It is evident that there are drastic variations in TEC values of Mt. Sinabung when it is erupting, and its value ranges from 15.5 TECU to 21.0 TECU, indicating the disturbance of the ionosphere due to the eruption. There are also drastic variations in TEC values at a distance of 100 km from the volcano, and its peak value is approximately 24.5 TECU, indicating the highest response of the ionosphere to the volcanic eruption. The TEC value at 300 km is moderately high and lies between 21.0 TECU and 24.5 TECU, indicating that the ionospheric perturbation due to the volcanic eruption still affects the ionosphere considerably at this location. Contrary to the situation in Mt. Kelud where there was a drastic fall in the influence of volcano effects with an increase in distance, the Mt. Sinabung explosion depicts that there is no drop in the enhancement of TEC regardless of how far we move from the volcano, indicating a higher degree of volcanic activity in relation to ionospheric perturbations. As illustrated above, there were ionospheric disturbances resulting from volcanic activities emanating from Mt. Sinabung up to 500 km away.

### 3.4. Prediction of TEC during volcanic eruption at Mt semeru (04-12-2021 to 06-12-2021)

Mount Semeru (Indonesian:*Gunung Semeru*) is an active volcano located in East Java, Indonesia. It is located in a subduction zone, where the Indo-Australian plate subducts under the Eurasian plate. It is the highest mountain on the island of Java. The name “Semeru” is derived from Meru, the central world mountain in Hinduism, or Sumeru, the abode of gods. This stratovolcano is Mahameru, meaning “The Great Mountain” in Sanskrit. It is one of the more popular hiking destinations in Indonesia.

Semeru’s eruptive history is extensive. Since 1818, at least 55 eruptions have been recorded (11 of which resulted in fatalities), consisting of lava and pyroclastic flows. All historical eruptions have had a Volcanic Explosivity Index (VEI) of 2 or 3. Semeru has been in a state of near-constant eruption from 1967 to the present. Mount Semeru saw a major eruption from December 4^th^ to December 6^th^, 2021, which led to massive volcanic activity and devastating effects. The eruption was initiated by the collapse of the summit lava dome, which emitted billowing clouds of superheated ash, tephra, soil, and debris that sped down the southeastern flank of the volcano. Pyroclastic flows, which are among the most lethal of volcanic hazards, were produced and travelled distances of several kilometers, destroying regions along the Kobokan drainage and adjacent areas.

Grey ash plumes, which were dense and rose up to 15 km into the air during this eruption, drifted in several directions, reducing visibility and blanketing large areas with volcanic ash. The eruption reached its peak on 6th December 2021 and created immense ionospheric perturbations, which were subsequently explored using TEC (Total Electron Content) changes. These perturbations also caused difficulties for communication networks and satellite-based navigation, necessitating comparative studies of predictive models like Hybrid ML, LightGBM, LSTM, MLR and the AR model.

The eruption had continued for around eleven days, resulting in massive destruction, deaths, and the evacuation of thousands of inhabitants. Ashfall had affected infrastructure, agriculture, and dwellings. Various zones were put on evacuation, and the Alert Level was increased by the Volcanological Survey of Indonesia (PVMBG).

The December 2021 Semeru eruption is one of the most significant volcanic catastrophes in recent years in Indonesia, both in the geophysical impact and the secondary ionospheric impacts that broke scientific predictive models.

We analyzed TEC variations during the volcanic eruption at Mount Semeru from November 20^th^ to December 15^th^, 2021 which showed its peak at December 4^th^ 2021, focusing on the performance of the Hybrid ML-DL Model compared with LightGBM, LSTM, MLR and the AR model, particularly at volcanic eruption from December 4thto December 6^th^, 2021 which had caused significant ionospheric disturbances, producing challenges in terms of the predictive accuracy of both models. This disaster occurred for a month in Indonesia. The performance metrics are summarised in Table 8.

**Table 8:** Performance evaluation and comparison during volcanic eruption at Mt. Semeru (20.11.2021 to 15.12.2021).

DATES	RMSE (TECU)					NRMSE					MBD (TECU)					RLE				
	Light GBM	LSTM	Hybrid	AR	MLR	Light GBM	LSTM	Hybrid	AR	MLR	Light GBM	LSTM	Hybrid	AR	MLR	Light GBM	LSTM	Hybrid	AR	MLR
11/20/2021	1.505	1.807	1.080	4.580	1.922	0.039	0.046	0.028	0.118	0.074	0.251	0.406	0.051	3.857	1.345	0.054	0.073	0.041	0.157	0.127
11/21/2021	2.230	2.480	1.804	6.772	2.348	0.052	0.058	0.042	0.159	0.113	0.680	1.082	0.630	6.015	1.169	0.139	0.107	0.083	0.574	0.163
11/22/2021	1.353	1.416	0.920	2.951	1.953	0.037	0.039	0.025	0.081	0.090	0.035	0.538	0.216	0.682	0.842	0.075	0.071	0.049	0.140	0.103
11/23/2021	1.521	1.432	1.212	3.604	1.299	0.039	0.036	0.031	0.091	0.057	0.050	0.590	0.304	0.322	0.047	0.079	0.075	0.059	0.196	0.097
11/24/2021	1.542	3.135	1.326	2.493	1.717	0.035	0.072	0.030	0.057	0.070	0.145	2.137	0.642	0.685	0.311	0.065	0.102	0.050	0.087	0.090
11/25/2021	1.587	2.392	1.226	4.473	2.196	0.035	0.053	0.027	0.100	0.086	0.282	1.592	0.234	0.391	0.054	0.060	0.078	0.034	0.194	0.123
11/26/2021	0.891	2.797	0.749	7.333	2.800	0.030	0.094	0.025	0.248	0.129	0.010	2.201	0.147	3.972	0.848	0.035	0.136	0.029	0.270	0.145
11/27/2021	1.243	1.828	1.067	7.572	1.915	0.028	0.041	0.024	0.170	0.072	0.362	0.539	0.186	4.016	1.078	0.058	0.084	0.047	0.232	0.082
11/28/2021	1.320	2.156	1.094	3.296	1.446	0.035	0.057	0.029	0.087	0.060	0.008	1.561	0.373	2.402	0.556	0.048	0.119	0.039	0.124	0.081
11/29/2021	1.160	1.686	0.969	2.677	2.119	0.037	0.053	0.031	0.085	0.090	0.240	0.181	0.135	1.066	0.043	0.041	0.065	0.031	0.083	0.098
11/30/2021	1.665	1.874	1.421	5.982	2.169	0.036	0.041	0.031	0.130	0.083	0.096	0.724	0.241	1.982	0.838	0.081	0.092	0.067	0.230	0.124
12/1/2021	1.367	1.951	1.097	4.683	1.868	0.034	0.049	0.027	0.117	0.078	0.362	0.848	0.088	1.274	0.502	0.057	0.091	0.040	0.177	0.085
12/2/2021	1.066	1.757	1.000	3.281	1.913	0.029	0.048	0.027	0.089	0.088	0.060	0.156	0.114	2.752	1.104	0.061	0.101	0.058	0.172	0.099
12/3/2021	1.136	2.077	1.002	7.092	2.756	0.059	0.108	0.052	0.370	0.154	0.158	0.473	0.075	4.998	1.220	0.055	0.100	0.049	0.264	0.141
12/4/2021	1.688	2.228	1.490	6.717	1.898	0.046	0.061	0.041	0.184	0.086	0.127	0.351	0.048	0.979	0.012	0.075	0.107	0.062	0.389	0.116
12/5/2021	0.990	0.990	0.679	2.250	1.345	0.028	0.028	0.019	0.063	0.063	0.027	0.079	0.091	1.645	0.708	0.036	0.057	0.026	0.121	0.087
12/6/2021	1.483	2.004	1.228	2.743	1.416	0.036	0.049	0.030	0.067	0.061	0.170	0.010	0.081	0.778	0.023	0.066	0.106	0.057	0.120	0.085
12/7/2021	1.197	1.236	0.894	2.068	1.426	0.032	0.033	0.024	0.055	0.064	0.109	0.198	0.036	1.091	0.876	0.049	0.066	0.037	0.099	0.072
12/8/2021	1.764	2.449	1.458	3.004	1.785	0.048	0.066	0.039	0.081	0.076	0.075	1.370	0.598	0.695	0.617	0.078	0.066	0.045	0.124	0.107
12/9/2021	0.983	1.364	0.872	4.578	2.546	0.034	0.047	0.030	0.157	0.128	0.233	0.110	0.113	3.377	1.544	0.050	0.076	0.043	0.152	0.112
12/10/2021	1.773	2.042	1.554	3.139	1.975	0.047	0.054	0.041	0.084	0.092	0.204	0.433	0.158	0.302	0.642	0.076	0.103	0.065	0.202	0.127
12/11/2021	1.127	1.321	0.861	6.043	2.477	0.042	0.049	0.032	0.223	0.140	0.341	0.787	0.261	4.583	1.571	0.065	0.092	0.054	0.270	0.112
12/12/2021	1.782	2.353	1.535	3.831	1.556	0.050	0.066	0.043	0.107	0.072	0.099	1.131	0.618	1.099	0.115	0.083	0.085	0.060	0.189	0.099
12/13/2021	1.716	2.378	1.560	3.969	2.916	0.045	0.063	0.041	0.105	0.136	0.154	1.062	0.054	0.874	1.206	0.063	0.087	0.048	0.195	0.141
12/14/2021	0.917	1.132	0.826	3.819	2.126	0.031	0.038	0.028	0.128	0.109	0.029	0.405	0.136	2.645	1.331	0.040	0.054	0.033	0.134	0.096
12/15/2021	1.503	2.098	1.206	3.477	1.811	0.043	0.059	0.034	0.099	0.121	0.134	0.996	0.364	2.261	0.862	0.057	0.066	0.038	0.111	0.081
**Average values during volcanic eruption days**	1.387	1.741	1.132	3.903	2.088	0.037	0.046	0.030	0.104	0.104	0.108	0.147	0.073	1.134	0.907	0.059	0.090	0.048	0.210	0.103
**Average values during Non-volcanic eruption days**	1.406	1.963	1.162	4.379	1.553	0.039	0.055	0.032	0.128	0.070	0.179	0.849	0.251	2.232	0.248	0.064	0.086	0.048	0.190	0.096
**Total** **Values**	1.329	1.738	1.162	3.702	2.056	0.041	0.054	0.036	0.124	0.094	0.110	0.510	0.190	1.681	0.812	0.056	0.078	0.045	0.148	0.110

By averaging the performance metrics, during the volcanic eruption, we analyzed that the ionosphere experienced considerable TEC deviations, with the Hybrid ML-DL Model achieving an RMSE of 1.132 TECU, not much higher than the LightGBM’s RMSE of 1.387 TECU, a little less than LSTM’s RMSE of 1.741 TECU, lower than MLR’s RMSE of 2.088 TECU and considerably lower than the AR model’s RMSE of 3.903 TECU.

The NRMSE for the Hybrid ML-DL Model was 0.030, compared to 0.037 for LightGBM, 0.046 for LSTM, 0.104 for MLR and 0.104 for AR, further emphasizing the Hybrid ML-DL Model’s superior accuracy. The MBD values also favoured the Hybrid ML-DL Model, averaging 0.108 TECU against the LightGBM’s 0.073 TECU, LSTM’s 0.147 TECU, MLR’s 0.907 TECU and AR model’s 1.134 TECU, which consistently underestimated TEC. The Hybrid ML-DL Model recorded an RLE of 0.048, significantly under-performing the LightGBM’s RLE of 0.059, LSTM ‘s RLE of 0.090, MLR’s RLE of 0.103 and AR model’s RLE of 0.210. Overall, the Hybrid ML-DL Model consistently demonstrated better accuracy among all metrics during this volcanic eruption compared to LightGBM, LSTM, MLR and AR Model.

**Fig 13:**
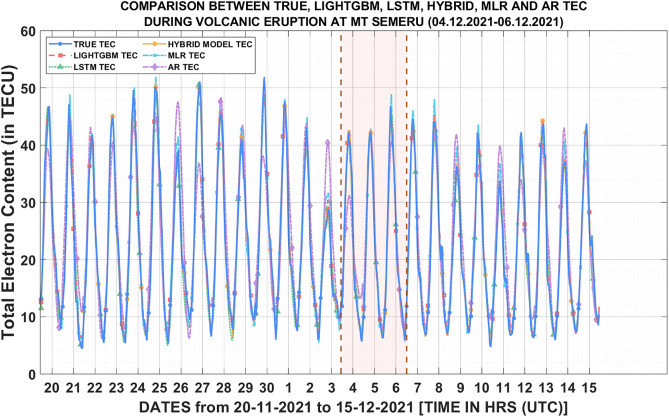
Comparison between TRUE, LightGBM, LSTM, Hybrid ML-DL Model, MLR and AR Model TEC during volcanic eruption at Mt. Semeru (04.12.2021 to 06.12.2021).

Fig 13 illustrates TEC variations from November 20^th^ to December 15^th^ 2021 and provides valuable information about the ionospheric response to the volcanic eruption and the models’ performance. The TEC and Hybrid ML-DL Model Predicted TEC curves show a close match and most accurately capture daily variations. On December 4 2021, during the volcanic eruption, around 08:00 (UTC), a peak TEC of 40.1603 TECU was observed due to the flare’s intensity. The Hybrid ML-DL Model closely predicted this at 40.128 TECU, whereas the LightGBM model with 39.641 TECU, the LSTM model around 40.98 TECU, the MLR model overestimated it at 41.642 TECU and the AR model underestimated it at 30 TECU. This emphasizes the ability of the Hybrid ML-DL Model to effectively capture the impacts of volcanic eruptions-induced ionospheric disruptions.

**Fig 14:**
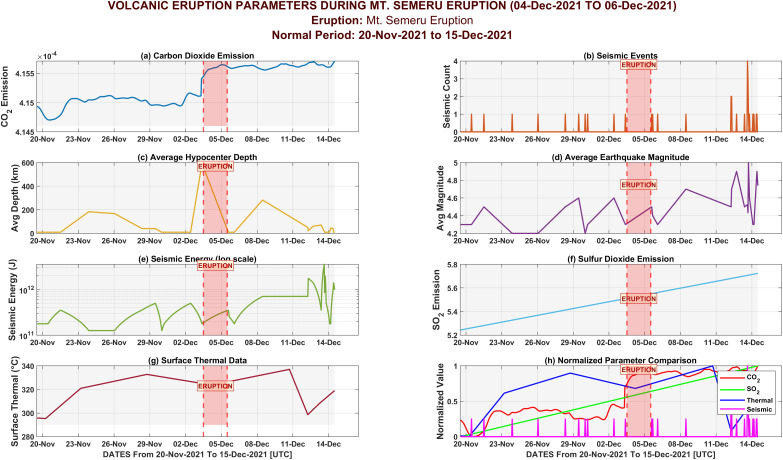
Eruption parameters analyzed during the volcanic eruption at Mt. Semeru (04.12.2021 to 06.12.2021).

Fig 14 shows the eruption parameters analyzed during the volcanic eruption at Mt. Semeru.

Still, there’s a clear witness of a sudden increase in True TEC during the noon of 6^th^ December 2021, but our Hybrid ML-DL Model could not predict the peak changes due to abrupt, unpredictable changes in CO2 and SO2 emissions. Even the AR model could not predict the peak caused in the True TEC values. So far, this is the most significant abrupt variation in the model; some other similar scenarios were captured on 30^th^ November 2021, which had the peak true TEC, and our ML model couldn’t predict them due to the increase in seismic activity.

**Fig 15:**
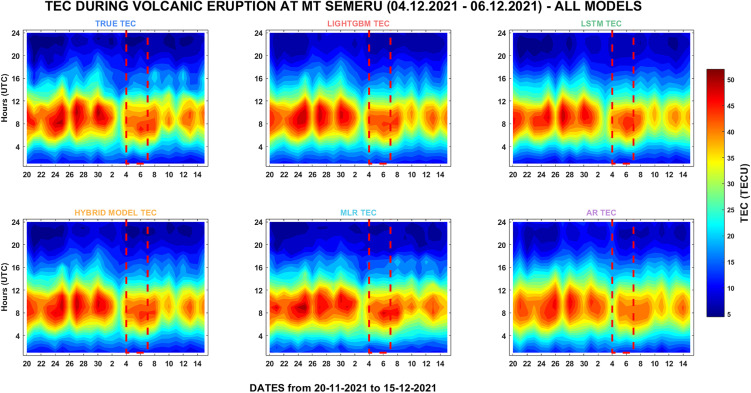
Comparison between True, Hybrid ML-DL Model, LightGBM, LSTM, MLR and AR TEC during volcanic eruption at Mt. Semeru as a contour plot.

Fig 15 shows the prediction of TEC variations during both normal and eruption dates as a contour plot, based on the minimum and maximum values among the True TEC, LightGBM, LSTM, AR Model, MLR Model, and the Hybrid ML-DL Model. By analyzing the contour plot, we can determine the TEC variations on an hourly basis by the variations in colour (Red for peak TECU, blue for low TECU).

On November 20^th^, 2021, the Hybrid ML-DL Model predicts the TECU correctly except at the peak due to some parametric variations.

By comparing the True TEC plot with the Hybrid model’s TEC plot, we can observe that both plots closely match each other, illustrating the superior accuracy of the Hybrid model in capturing the hourly variations in TEC during the volcanic eruption. The AR model’s plot shows a vast difference from the True TEC plot, which indicates that the AR fails in capturing the hourly TEC variations precisely. The MLR model has overestimated on most of the dates.

**Fig 16:**
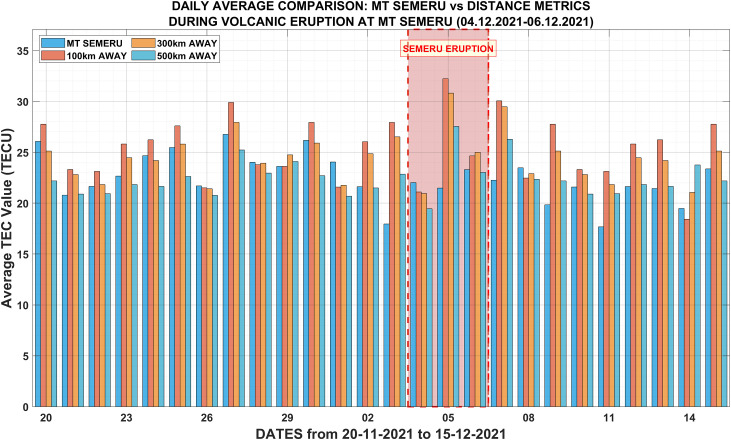
Comparison of daily average TEC during the volcanic eruption at Mt. Semeru across various distances.

Fig 16 depicts the daily average TEC values at Mt. Semeru and 3 different distances away (100 km, 300 km, and 500 km) from it in the period between November 20^th^ and December 15^th^, 2021. The section marked in red represents the time period during which the volcanic eruption was taking place (December 4–6, 2021). As shown in the graph above, it is seen that there are noticeable changes in the TEC values at Mt. Semeru during the volcanic eruption period. The TEC values lie in the range of 22.5 to 28.0 TECU, and this demonstrates that there are clear ionospheric disturbances due to the volcanic activity. Even at a distance of 100 km from Mt. Semeru, there are major fluctuations in TEC values with peaks up to around 27.0 TECU, thus demonstrating that there are ionospheric disturbances occurring closer to the volcanic eruption. At a distance of 300 km from Mt. Semeru, the TEC values are relatively high as well (20.5–26.0 TECU), and this indicates that there are disturbances of the ionosphere caused by the volcano. At a distance of 500 km from Mt. Semeru, the TEC values vary in the range of 19.5–24.0 TECU.

### 3.5. Prediction of TEC during volcanic eruption at Mt Ruang (16-04-2024 to 07-05-2024)

Ruang is the southernmost strato volcano in the Sangihe Islands arc, North Sulawesi, Indonesia. It comprises an island 4 by 5 kilometers wide. The summit contains a partial lava dome and reaches some 725 meters (2,379 ft) in altitude. At least 16 eruptions have been recorded from the volcano, with the first one occurring in 1808. It features a partial lava dome and is located at a geologically active zone prone to explosive volcanic eruptions. The volcano remained dormant for over two decades before its dramatic reawakening in April 2024. The activity began on April 16, 2024, at 21:45 local time (UTC – 8 hours). Initially, explosive eruptions occurred and over the next day, eruptions intensified; the alert level was escalated from level 3 to level 4. The eruption also caused potential collapse into the sea, as a result, tsunami warnings were also given. On 17 April, the eruptions emitted ash and sulphur dioxide plumes rising up to 15 kilometer above the summit according to Copernicus monitoring. On April 30, the ash plumes rose up to 19.2 kilometer, marking the peak of the volcano’s eruptive phase. These plume heights were confirmed by satellite and atmospheric monitoring. This eruption caused the closure of all international and local airports in the surrounding area. Approximately 800 residents were evacuated from Ruang and moved to nearby Tagulandang Island. The higher authorities issued tsunami alerts, as the collapse of eruptive material into the sea could generate high waves. The area around the volcano was kept under high alert due to continuous seismicity and ash emissions. According to the reports, almost 501 houses and buildings on the neighbouring islands were damaged due to continuous ash fall, debris, and seismic shaking. By April 22, the alert status was downgraded from level 4 to level 3. By early May 2024, the eruptive activities had declined to low levels. Although no formal reports were issued, the eruption had clearly weakened by early May. After the April 30 explosion, no further major explosions were recorded, and the authorities also reduced the emergency measures.

We analyzed TEC variations during the volcanic eruption at Mount Ruang from April 9^th^ to May 8^th^ 2024, which showed its peak at April 16^th^ 2024, focusing on the performance of the Hybrid ML-DL Model compared with LightGBM, LSTM, MLR and the AR model, particularly at the volcanic eruption from April 16^th^ to May 7^th^ 2024, caused significant ionospheric disturbances, challenging the predictive accuracy of both models. This disaster occurred for almost 22 days in Indonesia. The performance metrics, summarized in Table 9, reveal the superior accuracy of the Hybrid ML-DL Model across all parameters. By averaging the performance metrics, during the volcanic eruption, we analyzed that the ionosphere experienced considerable TEC deviations, with the Hybrid ML-DL Model achieving an RMSE of 2.841 TECU significantly lower than the LightGBM’s RMSE of 3.484 TECU, LSTM’s RMSE of 5.084 TECU, MLR Model’s RMSE of 5.345 TECU and AR model’s RMSE of 6.285 TECU. The NRMSE for the Hybrid ML-DL Model was 0.03 compared to 0.036 for LightGBM, 0.053 for LSTM,0.089 for MLR and 0.066 for AR, further emphasizing the Hybrid ML-DL Model’s superior accuracy. The MBD values also favored the Hybrid ML-DL Model, averaging 0.546 TECU against the LightGBM’s 0.546 TECU, LSTM’s 1.73 TECU, MLR of 2.16 TECU and AR model’s 2.072 TECU, which consistently underestimated TEC. The Hybrid ML-DL Model recorded an RLE of 0.069, significantly under-performing the LightGBM’s RLE of 0.093, LSTM ‘s RLE of 0.148, MLR of 0.174 and AR model’s RLE of 0.129. Overall, the Hybrid ML model consistently demonstrated better accuracy across all metrics during this volcanic eruption compared to LightGBM, LSTM, MLR and AR Model.

**Table 9:** Performance evaluation and comparison during volcanic eruption at Mt. Ruang (09.04.2024 to 08.05.2024).

DATES	RMSE (TECU)					NRMSE					MBD (TECU)					RLE				
	Light GBM	LSTM	Hybrid	AR	MLR	Light GBM	LSTM	Hybrid	AR	MLR	Light GBM	LSTM	Hybrid	AR	MLR	Light GBM	LSTM	Hybrid	AR	MLR
4/09/2024	2.443	3.959	2.166	6.140	3.453	0.026	0.043	0.023	0.067	0.052	0.734	2.068	0.297	1.298	0.570	0.075	0.114	0.069	0.096	0.086
4/10/2024	3.266	6.456	2.308	6.388	3.276	0.032	0.063	0.023	0.062	0.051	0.067	1.125	0.322	3.408	1.107	0.144	0.326	0.123	0.160	0.083
4/11/2024	3.846	6.343	2.801	5.483	3.588	0.040	0.066	0.029	0.057	0.058	0.021	3.052	0.475	0.834	1.810	0.074	0.191	0.050	0.085	0.106
4/12/2024	4.226	4.831	3.754	4.780	5.651	0.043	0.049	0.038	0.048	0.099	0.824	1.116	0.560	0.515	3.599	0.091	0.145	0.083	0.095	0.156
4/13/2024	4.076	4.989	3.637	6.697	5.055	0.044	0.054	0.040	0.073	0.092	0.855	1.392	1.000	4.113	2.829	0.066	0.067	0.048	0.129	0.161
4/14/2024	3.204	4.838	2.321	5.940	4.428	0.032	0.048	0.023	0.059	0.083	0.322	1.348	0.519	0.706	3.212	0.057	0.140	0.047	0.135	0.140
4/15/2024	3.497	5.226	3.267	5.725	3.725	0.038	0.056	0.035	0.062	0.072	0.376	2.085	0.883	0.990	1.782	0.063	0.089	0.048	0.123	0.191
4/16/2024	3.746	6.773	3.546	8.402	4.234	0.036	0.065	0.034	0.081	0.082	0.573	1.077	0.252	3.516	1.168	0.112	0.213	0.103	0.206	0.084
4/17/2024	3.978	5.919	3.666	7.041	3.922	0.040	0.059	0.037	0.070	0.072	0.544	1.284	0.703	2.279	0.557	0.073	0.122	0.062	0.170	0.135
4/18/2024	4.075	6.029	3.819	7.229	3.153	0.041	0.060	0.038	0.072	0.055	0.560	1.201	0.739	2.358	0.035	0.071	0.113	0.059	0.179	0.140
4/19/2024	4.306	7.622	3.740	12.393	6.378	0.046	0.081	0.040	0.132	0.118	1.402	1.892	0.903	8.216	3.375	0.061	0.109	0.050	0.224	0.260
4/20/2024	6.859	12.456	5.952	15.398	5.330	0.066	0.120	0.057	0.148	0.101	1.756	6.886	0.307	1.899	4.779	0.111	0.304	0.100	0.269	0.196
4/21/2024	5.573	9.742	5.047	11.189	4.257	0.053	0.092	0.048	0.106	0.076	0.391	5.542	0.951	2.242	1.672	0.099	0.194	0.088	0.170	0.278
4/22/2024	4.577	8.482	4.498	6.452	4.187	0.041	0.075	0.040	0.057	0.073	0.331	2.279	0.025	0.466	0.764	0.086	0.246	0.084	0.130	0.115
4/23/2024	4.133	5.361	3.467	5.883	4.911	0.038	0.049	0.032	0.054	0.085	1.108	2.049	0.267	3.700	2.338	0.074	0.106	0.065	0.085	0.193
4/24/2024	3.505	9.303	3.104	10.350	5.601	0.031	0.081	0.027	0.090	0.090	0.551	6.086	0.562	6.916	0.118	0.064	0.319	0.061	0.240	0.109
4/25/2024	3.747	4.913	2.949	7.391	3.759	0.037	0.049	0.029	0.074	0.061	0.288	2.097	0.655	4.105	0.734	0.057	0.084	0.038	0.129	0.155
4/26/2024	3.607	8.549	3.183	12.461	4.572	0.034	0.081	0.030	0.118	0.075	1.059	7.340	1.874	9.120	0.637	0.140	0.350	0.129	0.600	0.104
4/27/2024	5.420	17.810	5.111	8.281	6.644	0.054	0.178	0.051	0.083	0.092	1.867	17.064	3.145	5.586	5.185	0.149	0.786	0.147	0.150	0.152
4/28/2024	3.861	19.429	3.861	7.208	6.968	0.045	0.224	0.045	0.083	0.097	2.107	18.915	2.107	5.549	1.341	0.093	0.734	0.093	0.136	0.163
4/29/2024	4.159	24.671	4.159	4.446	6.578	0.047	0.278	0.047	0.050	0.092	1.477	24.242	1.477	0.911	1.617	0.174	1.182	0.174	0.193	0.178
4/30/2024	5.123	10.097	5.097	12.603	6.999	0.074	0.146	0.074	0.182	0.093	2.911	8.853	3.088	9.444	3.926	0.152	0.329	0.151	0.228	0.146
5/1/2024	4.547	10.937	4.450	12.330	6.652	0.071	0.170	0.069	0.192	0.097	0.142	1.180	0.511	4.962	0.336	0.094	0.260	0.089	0.209	0.197
5/2/2024	4.063	7.344	3.916	9.069	4.343	0.058	0.104	0.056	0.129	0.060	1.892	5.431	2.315	2.358	1.894	0.108	0.286	0.104	0.289	0.079
5/3/2024	4.210	6.161	3.834	10.937	6.954	0.047	0.068	0.043	0.121	0.111	0.389	2.314	0.971	6.112	3.776	0.270	0.411	0.263	0.256	0.341
5/4/2024	3.781	4.180	3.058	6.035	6.345	0.042	0.047	0.034	0.068	0.111	0.255	1.495	0.657	0.604	3.908	0.087	0.110	0.069	0.109	0.323
5/5/2024	3.366	8.290	3.084	12.533	4.833	0.035	0.087	0.032	0.131	0.093	0.201	2.642	0.239	0.072	2.754	0.100	0.163	0.076	0.392	0.097
5/6/2024	3.315	4.028	2.472	9.127	4.685	0.035	0.043	0.026	0.097	0.092	0.424	1.651	0.311	4.712	2.280	0.174	0.111	0.082	0.212	0.194
5/7/2024	3.798	3.442	2.972	10.935	6.298	0.048	0.043	0.037	0.138	0.147	1.085	0.876	0.647	3.736	4.345	0.106	0.109	0.074	0.189	0.209
5/8/2024	4.272	10.285	3.929	10.866	6.214	0.051	0.122	0.047	0.130	0.128	1.159	7.359	1.372	4.804	2.313	0.143	0.380	0.122	0.267	0.413
**Total** **Values**	4.332	9.703	3.977	9.382	6.423	0.047	0.106	0.043	0.102	0.110	0.990	5.993	1.088	4.021	2.819	0.109	0.321	0.100	0.218	0.239
**Average values during volcanic eruption days**	3.484	5.084	2.841	6.285	5.345	0.036	0.053	0.030	0.066	0.089	0.453	1.730	0.546	2.072	2.160	0.093	0.148	0.069	0.129	0.174
**Average values during Non-volcanic eruption days**	3.477	1.427	2.757	11.290	4.167	0.042	0.021	0.034	0.133	0.072	0.271	2.797	0.132	2.832	2.129	0.136	0.021	0.089	0.273	0.131

**Fig 17:**
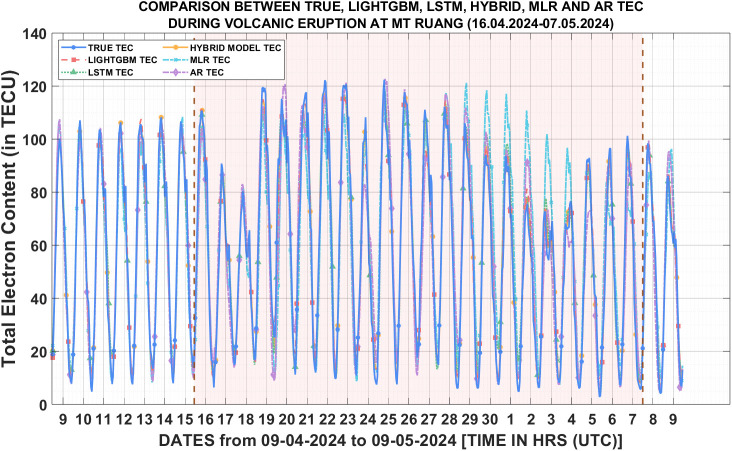
Comparison between TRUE, LightGBM, LSTM, Hybrid ML-DL Model, MLR and AR TEC during volcanic eruption at Mt. Ruang (16.04.2024 to 07.05.2024).

**Fig 18:**
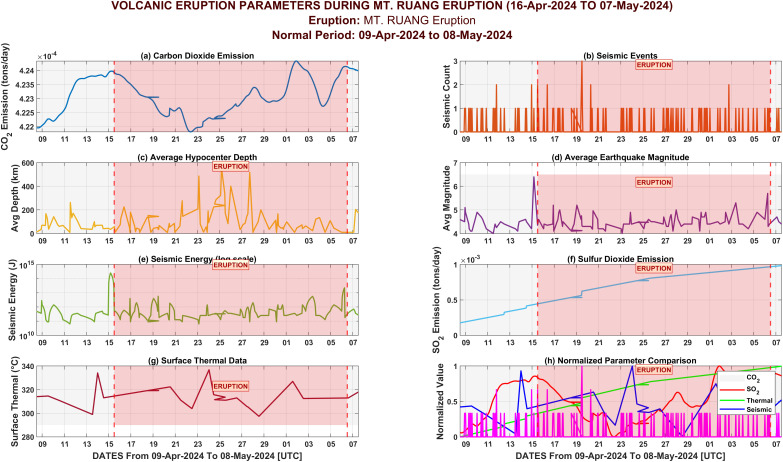
Eruption parameters analyzed during the volcanic eruption at Mt. Ruang (16.04.2024 to 07.05.2024).

Fig 17. illustrates TEC variations from April 9^th^ to May 8^th^ 2024, and provides valuable insights into the ionospheric response to the volcanic eruption and the models’ performances. The TEC and Hybrid ML-DL Model Predicted TEC curves show a close match, accurately capturing daily variations. On May 1st, 2024, during the volcanic eruption, around 08:00 (UTC), a peak TEC of 78 TECU was observed due to the eruption. The Hybrid ML-DL Model closely predicted this at 81 TECU, whereas the LightGBM model with 84 TECU, the LSTM model around 87 TECU, MLR model of 89 TECU and the AR model underestimated it at 96 TECU. This highlights the Hybrid ML-DL Model’s ability to effectively capture the impacts of a rise in TEC due to an increase in plasma bubbles during volcanic eruption, and MLR fails to predict the TEC, resulting in high deviation compared to other models during eruption. With the continuation, we can see that due to the explosion at Mt. Ruang on 16^th^ April 2024, there was an increase in Plasma bubble, which tends to have an unexpected increase in TEC in the range of 100–110 TECU during noon in the upcoming days. Here, with the help of Hybridization of ML models, we can predict these unexpected changes up to the marginal level compared to other models based on hyper-tuning and training. Fig 18 shows the eruption parameters analyzed during the volcanic eruption at Mt.Ruang.

Still, there’s a clear witness of a sudden increase in True TEC during the night of 19^th^ April, but our Hybrid ML-DL Model could not predict the peak changes because of the abrupt increase in seismic activity. Even the AR model could not predict the peak caused in the True TEC values. Likewise in April 29^th^ and May 1^st^, there had also been sudden events caused by variations in CO2 and SO2 emission levels, which also could not be predicted by the Hybrid ML-DL Model and the other models.

**Fig 19:**
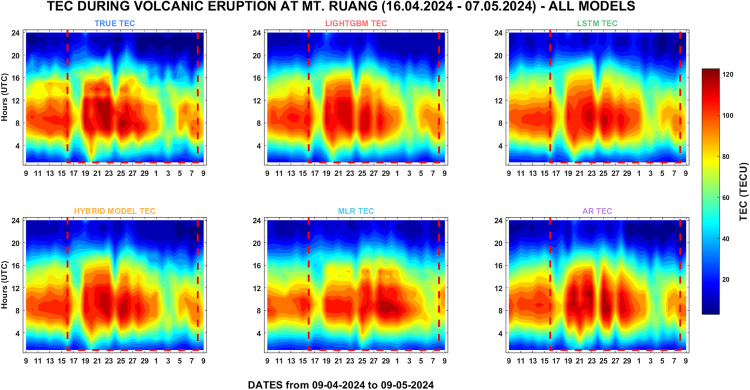
Comparison between TRUE, LightGBM, LSTM, Hybrid ML-DL Model, MLR and AR Model TEC during volcanic eruption at Mt. Ruang (16.04.2024 to 07.05.2024) using Contour plot.

Here, Fig 19 represents the contour plot of TRUE TEC, LightGBM MLR and AR TEC during both normal and eruption dates. Based on the maximum value and minimum value between TRUE TEC, Hybrid ML-DL Model, LightGBM, LSTM, MLR and AR Model TEC. By analyzing the contour plot, we can determine the TECU as regions in the particular hours of the day, in the variation in colour (red for peak TECU, blue for low TECU).

During 19th April to 27th April 2024, the Hybrid ML-DL Model predicts the TECU values correctly except at the peak due to the eruption parametric changes. On comparing with the TRUE TEC contour plot, the Hybrid ML-DL Model predicted TEC replicates it, as it matches almost all the regions with maximum accuracy. Here, the AR model doesn’t match with TRUE TEC, which indicates there is a slight difference between the TECU when comparing them in each hour (UTC).

**Fig 20:**
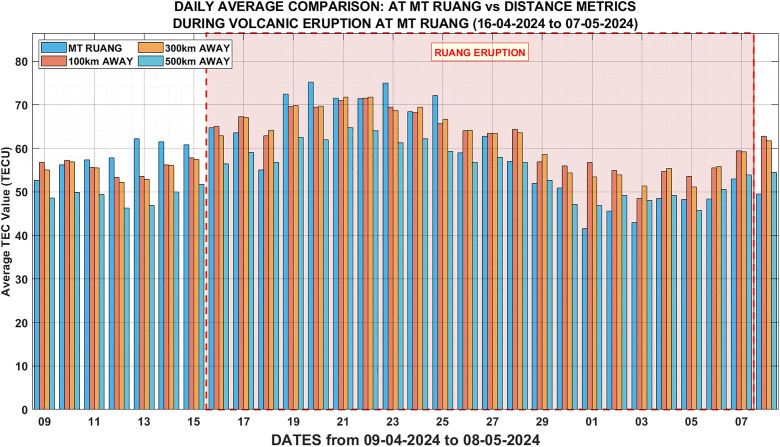
Comparison of daily average TEC during volcanic eruption at Mt. Ruang across various distances.

Fig 20 shows the daily average value of TEC measured at Mt. Ruang and the three distances from Mt. Ruang (100 km, 300 km, and 500 km). It can be seen that the time between April 16 and May 7, 2024, is marked with the shaded red region representing the volcanic eruption period, during which severe ionospheric disturbances were witnessed. It has been noticed that during the period of volcanic eruptions, there are noticeable changes in the TEC values at Mt. Ruang, which increase progressively from 58.0 to 101.0 TECU, implying that severe ionospheric disturbances have occurred due to volcanic eruptions. It is clear that significant disturbances have also occurred in the TEC values at 100 km distance from the volcano since the TEC values at this location peaked at 100.0 TECU, which is almost identical to the values at Mt. Ruang. The TEC values at 300 km distance have consistently remained high during the eruption (57.0–99.0 TECU) and demonstrate that disturbances in the ionosphere caused by volcanic eruptions propagate far into space with great force. The same increasing trend is observed at 500 km distance (55.0–97.0 TECU).

### 3.6. Prediction of TEC during volcanic eruption at Mt Etna (15.11.2013, 16.11.2013, 17.11.2013, 23.11.2013, 28.11.2013 and 02.12.2013)

Mount Etna is a tall volcano that sits on the eastern side of Sicily, which is an island in Italy. It reaches a peak height of roughly 3,329 meters (10,922 feet), but the height can change often because the volcano is still active and keeps erupting. It is famous for its ongoing eruptions, which can range from gentle strombolian activity to stronger, more explosive events. Mount Etna is seen as one of the most active volcanoes in the world because it often erupts, and during these eruptions, it sends up lava fountains, ash clouds, and volcanic gases. Many eruptions have been recorded throughout history, which makes it one of the most closely studied volcanoes in the world.

The volcano was active during November and December 2013, with eruptions happening on November 15^th^, 16^th^, 17^th^, 23^rd^, 28^th^, and December 2^nd^. These eruptions had strong lava shooting high into the air, released a lot of volcanic ash and gases, and created thick clouds of ash that went up very high into the sky. The eruptions were at their strongest during these dates, creating intense heat and sending out a lot of volcanic material. The ash clouds spread to nearby areas, leading to some small problems and brief issues with flights in the surrounding regions.

The eruptive activity also caused disturbances in the atmosphere, like sound waves and gravity waves, which travelled upward into the ionosphere. These waves helped to disrupt the ionospheric electron density, causing clear changes in the TEC. Although the eruptions weren’t as big or explosive as some major volcanic events, they were strong enough to cause noticeable changes in the ionosphere, which can mess up satellite-based navigation and communication systems.

We looked at how the TEC changed during the volcanic eruption at Mount Etna from November 15^th^ to December 15^th^, 2013. The biggest changes in TEC happened on the days when the eruption was taking place. We compared how well the Hybrid ML-DL Model performed against other models such as LightGBM, LSTM, MLR, and AR, especially during the days when the eruption was happening. The frequent eruptions during this time led to some ionospheric issues, making it harder for the models to predict accurately. These disturbances occurred from time to time over several days, indicating that Mount Etna remained very active.

By looking at the average performance results from Table 10, during the volcanic eruption, we found that the ionosphere had clear changes in TEC levels. The Hybrid ML-DL Model showed smaller RMSE and NRMSE values than the LightGBM, LSTM, MLR and AR models, which means it works better at making predictions. The MBD values also supported the Hybrid ML-DL Model, indicating very little bias in its predictions, while the AR model kept underestimating TEC changes because it relied only on past values in a straight-line way. The Hybrid ML-DL Model also had lower RLE values, showing better reliability in understanding how the ionosphere changes over time. Overall, the Hybrid ML model kept showing higher accuracy in all the measurements during this volcanic eruption when compared to the LightGBM, LSTM, MLR and AR models.

**Table 10:** Performance evaluation and comparison during volcanic Eruption at Mt. Etna (15.11.2013 to 15.12.2013).

DATES	RMSE (TECU)					NRMSE					MBD (TECU)					RLE				
	Light GBM	LSTM	Hybrid	AR	MLR	Light GBM	LSTM	Hybrid	AR	MLR	Light GBM	LSTM	Hybrid	AR	MLR	Light GBM	LSTM	Hybrid	AR	MLR
11/15/2013	1.300	1.642	1.129	3.152	2.259	0.051	0.064	0.044	0.123	0.116	0.357	0.891	0.391	1.227	1.046	0.060	0.077	0.049	0.144	0.130
11/16/2013	0.941	1.750	0.792	2.668	2.045	0.029	0.055	0.025	0.083	0.106	0.131	0.485	0.125	0.154	0.498	0.056	0.113	0.044	0.137	0.120
11/17/2013	1.173	1.404	1.046	2.059	2.139	0.035	0.042	0.031	0.062	0.119	0.275	0.637	0.328	1.237	0.230	0.059	0.092	0.057	0.132	0.136
11/18/2013	1.745	1.888	1.566	2.789	1.715	0.065	0.070	0.058	0.104	0.104	0.920	1.367	0.985	2.109	0.577	0.140	0.164	0.132	0.199	0.136
11/19/2013	1.209	1.472	0.980	2.586	1.506	0.046	0.056	0.038	0.099	0.094	0.548	0.837	0.486	1.749	0.014	0.088	0.086	0.064	0.161	0.128
11/20/2013	1.201	1.618	1.164	2.689	1.727	0.043	0.058	0.042	0.097	0.096	0.174	0.337	0.202	0.735	0.368	0.048	0.086	0.045	0.125	0.111
11/21/2013	1.147	1.474	1.105	3.178	1.239	0.044	0.057	0.043	0.123	0.080	0.676	1.071	0.694	2.827	0.357	0.095	0.121	0.090	0.244	0.101
11/22/2013	1.401	1.894	1.133	2.695	1.300	0.060	0.081	0.048	0.115	0.086	0.276	1.440	0.854	1.735	0.563	0.092	0.121	0.078	0.159	0.108
11/23/2013	1.679	1.987	1.458	3.146	2.625	0.057	0.067	0.049	0.106	0.152	0.242	0.326	0.323	0.874	0.390	0.075	0.089	0.062	0.124	0.135
11/24/2013	1.251	1.429	1.017	4.761	1.471	0.067	0.076	0.054	0.253	0.101	0.323	0.569	0.296	3.503	0.352	0.071	0.087	0.059	0.232	0.098
11/25/2013	1.132	1.739	1.009	2.516	1.811	0.064	0.099	0.057	0.143	0.132	0.270	0.776	0.290	2.201	0.422	0.053	0.076	0.043	0.175	0.121
11/26/2013	1.358	2.617	1.335	2.740	1.032	0.075	0.144	0.073	0.151	0.081	0.744	2.088	0.831	2.565	0.331	0.105	0.183	0.102	0.265	0.104
11/27/2013	1.155	1.986	0.952	1.575	1.019	0.082	0.141	0.068	0.112	0.083	0.543	1.228	0.544	1.372	0.073	0.087	0.113	0.065	0.124	0.105
11/28/2013	1.530	2.360	1.239	2.199	1.205	0.110	0.170	0.089	0.158	0.104	0.731	1.784	0.883	1.990	0.087	0.132	0.174	0.112	0.215	0.124
11/29/2013	1.612	2.616	1.248	3.124	1.384	0.081	0.131	0.063	0.157	0.103	0.001	2.178	0.594	1.086	0.457	0.114	0.187	0.101	0.171	0.116
11/30/2013	0.855	1.210	0.692	1.018	1.220	0.043	0.060	0.034	0.051	0.087	0.178	0.048	0.045	0.237	0.509	0.052	0.074	0.041	0.071	0.114
12/1/2013	1.217	1.650	1.081	3.261	1.669	0.048	0.065	0.043	0.129	0.105	0.177	0.736	0.309	1.394	0.669	0.066	0.079	0.052	0.132	0.106
12/2/2013	1.175	1.598	1.139	4.789	1.548	0.077	0.104	0.074	0.313	0.126	0.703	0.530	0.682	3.910	0.807	0.117	0.149	0.111	0.321	0.111
12/3/2013	1.497	2.013	1.282	2.313	1.446	0.080	0.108	0.069	0.124	0.117	0.571	1.216	0.444	0.153	0.599	0.124	0.200	0.106	0.171	0.121
12/4/2013	1.046	1.308	0.909	1.537	1.570	0.055	0.069	0.048	0.081	0.122	0.375	0.332	0.278	0.433	0.153	0.063	0.068	0.045	0.097	0.112
12/5/2013	1.003	1.424	0.939	1.588	1.314	0.058	0.082	0.054	0.092	0.097	0.099	0.501	0.119	0.157	0.319	0.051	0.091	0.047	0.099	0.108
12/6/2013	0.805	1.404	0.694	1.538	1.354	0.038	0.067	0.033	0.073	0.098	0.127	0.254	0.023	0.219	0.131	0.037	0.075	0.030	0.103	0.088
12/7/2013	0.527	0.755	0.397	1.124	0.877	0.027	0.038	0.020	0.057	0.062	0.039	0.081	0.055	0.101	0.114	0.030	0.045	0.022	0.064	0.079
12/8/2013	1.001	1.812	0.759	6.890	2.546	0.030	0.054	0.023	0.206	0.133	0.154	0.654	0.247	4.878	1.286	0.047	0.085	0.038	0.198	0.111
12/9/2013	0.683	1.125	0.573	4.709	1.167	0.031	0.051	0.026	0.213	0.077	0.113	0.763	0.269	3.338	0.535	0.046	0.085	0.039	0.210	0.085
12/10/2013	0.982	1.151	0.806	2.690	1.353	0.038	0.044	0.031	0.104	0.085	0.079	0.186	0.089	0.907	0.543	0.047	0.076	0.038	0.097	0.094
12/11/2013	1.029	1.320	0.945	1.528	1.263	0.044	0.057	0.041	0.066	0.084	0.261	0.710	0.356	0.676	0.321	0.063	0.095	0.057	0.087	0.110
12/12/2013	1.275	1.665	1.135	1.729	1.829	0.048	0.063	0.043	0.066	0.123	0.100	0.686	0.198	0.291	0.349	0.065	0.110	0.057	0.090	0.116
12/13/2013	1.422	1.408	1.188	1.550	1.703	0.062	0.062	0.052	0.068	0.117	0.095	0.119	0.012	0.216	0.381	0.073	0.078	0.054	0.093	0.119
12/14/2013	1.317	1.916	1.198	5.223	2.159	0.047	0.068	0.043	0.186	0.116	0.347	0.931	0.407	3.917	0.726	0.054	0.080	0.044	0.174	0.113
12/15/2013	1.192	1.506	1.021	2.973	0.575	0.040	0.050	0.034	0.099	0.065	0.517	0.827	0.354	2.173	0.575	0.073	0.113	0.063	0.236	0.063
**Total Values**	1.040	1.453	0.889	2.876	1.551	0.049	0.068	0.042	0.125	0.104	0.188	0.551	0.191	1.612	0.475	0.059	0.093	0.048	0.137	0.128
**Average values during volcanic eruption days**	1.300	1.790	1.134	3.002	1.953	0.060	0.084	0.052	0.141	0.098	0.406	0.775	0.455	1.565	0.437	0.083	0.116	0.073	0.179	0.016
**Average values during** **Non-volcanic eruption days**	1.163	1.608	1.007	2.707	1.454	0.052	0.073	0.045	0.117	0.102	0.307	0.791	0.358	1.547	0.44	0.071	0.103	0.060	0.150	0.11

**Fig 21:**
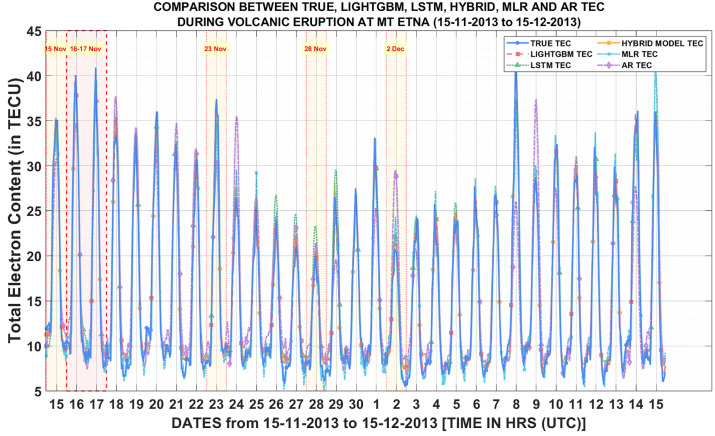
Comparison between TRUE, LightGBM, LSTM, Hybrid ML-DL Model, MLR and AR TEC during volcanic eruption at Mt. Etna (15.11.2013, 16.11.2013, 17.11.2013, 23.11.2013, 28.11.2013 and 02.12.2013).

**Fig 22:**
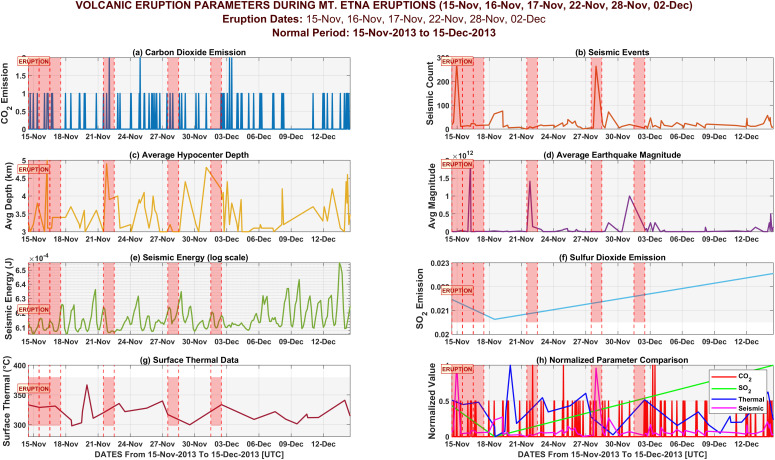
Eruption parameters analyzed during volcanic eruption at Mt. Etna (15.11.2013, 16.11.2013, 17.11.2013, 23.11.2013, 28.11.2013 and 02.12.2013).

Fig 21. illustrates TEC variations from November 15^th^ to December 15^th^, 2013, and provides valuable insights into the ionospheric response to the volcanic eruption and the models’ performances. The TEC and Hybrid ML-DL Model Predicted TEC curves show a close match, accurately capturing daily variations. On November 16^th^, 2013, during the volcanic eruption, around 11:00 (UTC), a peak TEC of 41 TECU was observed due to the flare’s intensity. The Hybrid ML-DL Model closely predicted this at 38 TECU, whereas the LightGBM model with 38 TECU, the LSTM model around 37 TECU, the MLR and the AR model underestimated it at 37.3 and 35 TECU respectively, which is due to the increase in seismic activity from 15^th^ November to 17^th^ November 2013 and there is a significant increase in CO2 and SO2 emissions. On November 23^rd^, 2013, during the volcanic eruption, around 12:00 (UTC), a peak TEC of 37.5 TECU was observed due to the flare’s intensity. The Hybrid ML-DL Model closely predicted this at 36.5 TECU, whereas the LightGBM model with 36 TECU, the LSTM model around 35 TECU, the MLR model overestimated it at 35.3 TECU and the AR model underestimated it at 30 TECU. On November 28^th^, 2013, during the volcanic eruption, around 10:00 (UTC), a peak TEC of 20 TECU was observed due to the flare’s intensity. The Hybrid ML-DL Model closely predicted this at 20.5 TECU, whereas the LightGBM model at 18 TECU, the LSTM model at around 23 TECU, and the MLR and AR models underestimated it at 18.8 and 21 TECU, respectively. On December 2^nd^, 2013, during the volcanic eruption, around 12:00 (UTC), a peak TEC of 21 TECU was observed due to the flare’s intensity. The Hybrid ML-DL Model closely predicted this at 22 TECU, whereas the LightGBM model with 20 TECU, the LSTM model around 37 TECU, the MLR model around 21.8 TECU and the AR model overestimated it at 29 TECU. Here, the Hybrid ML-DL Model tends to predict the change in TECU correctly as compared with other individual models and also concludes that the AR model makes little worse to predict the changes, as it follows a pattern in predicting TEC in all the days, unable to predict the change in TECU due to these parametric changes due to this volcanic eruption as they make predictions based only on TEC values. Fig 22 shows the Eruption Parameters analyzed during the volcanic eruption at Mt. Etna.

Still, there’s a clear witness of a sudden increase in True TEC during 24th November, but our Hybrid ML-DL Model could not predict the peak changes because of abrupt, unpredictable changes in eruption parameters. Even the AR model could not predict the peak caused in the True TEC values. Likewise, on November 23^rd^ and December 2^nd^ and 9^th^, 2013, there had also been sudden events caused by variations in the parameters, which also could not be predicted by the Hybrid ML-DL Model and the other model.

**Fig 23:**
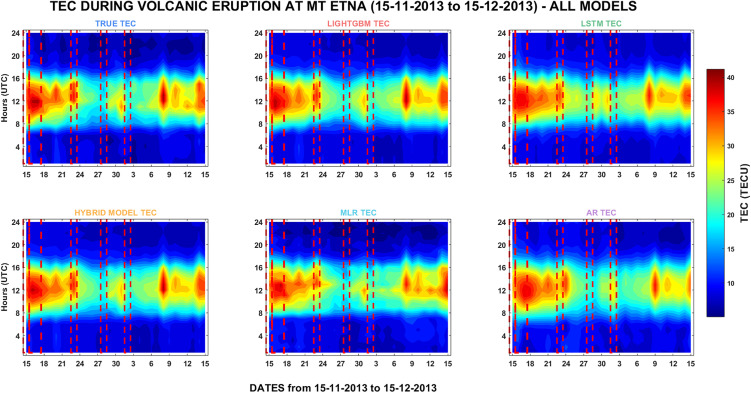
Comparison between TRUE, LightGBM, LSTM, Hybrid ML-DL Model, MLR model and AR Model TEC during volcanic eruption at Mt Etna (16.04.2024 to 07.05.2024) using Contour plot.

Here, Fig 23 represents the contour plot of TRUE TEC, LightGBM and AR TEC during both normal and SF dates. Based on the maximum value and minimum value between TRUE TEC, Hybrid ML-DL Model, LightGBM, LSTM, MLR and AR TEC. By analyzing the contour plot, we can determine the TECU as regions in the particular hours of the day, in the variation in colour (red for peak TECU, blue for low TECU).

During November 16^th^ and 17^th^ 2013, the Hybrid ML-DL Model predicts the TECU values correctly except at the peak due to the parametric changes. On comparing with the TRUE TEC contour plot, the Hybrid ML-DL Model predicted TEC replicates it, as it matches almost all the regions with maximum accuracy. The MLR model failed to predict the peaks very closely. Here, the AR model does not match with TRUE TEC, which indicates there is a slight difference between the TECU when comparing them in each hour (UTC).

**Fig 24:**
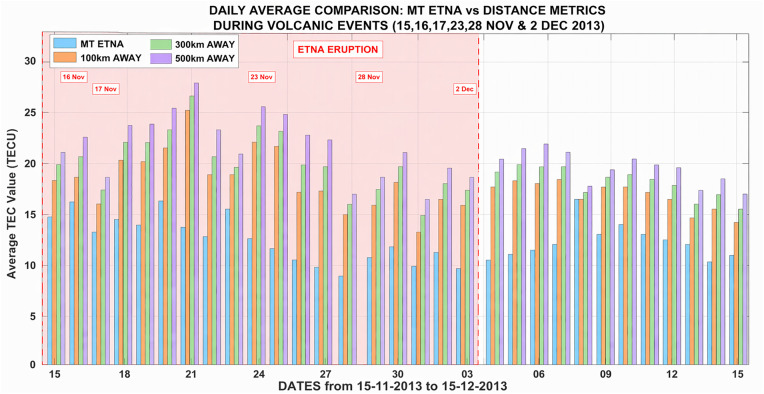
Comparison of daily average TEC during volcanic eruption at Mt. Etna across various distances.

Fig 24 above illustrates the daily average TEC values recorded at Mt. Etna and the three distances away (100 km, 300 km, and 500 km) from it for the months of November and December 2013. The area marked by the shaded red region illustrates the duration of the volcanic eruption (15 November–2 December). It is observed that there are drastic changes in the TEC values for Mt. Etna during the time interval, indicating disturbance of the ionosphere due to volcanic eruption. There are significant variations in the TEC values even at a distance of 100 km from the volcano. However, the changes become more pronounced with an increase in the distance from the volcanic source. Hence, at a distance of 100 km from the volcano, the changes in the TEC values become very significant, while at 500 km distance, they remain relatively constant. The above illustration clearly shows how the effect of a volcanic eruption decreases sharply with distance, thus causing minimum interference with the ionosphere farther from the volcano.

### 3.7. Prediction of TEC during volcanic eruption at Mt Lasoufriere (09-04-2021 to 22-04-2021)

Mount La Soufriere is an active stratovolcano located on the island of Saint Vincent in the Caribbean. It has an elevation of about 1,234 meters (4,049) and it is the highest peak in Saint Vincent and the Grenadines. Mount La Soufriere is known for its highly explosive eruptions, characterized by the emission of volcanic ash, gases, and pyroclastic material, which has a significant impact on both the atmosphere and the surrounding environment. The volcano has a long history of eruptions, with major eruptions recorded in 1718, 1812, 1902, and the most recent eruption that took place in April 2021.

The April 2021 eruption is considered one of the most significant volcanic eruptions in the Caribbean in recent decades. The eruption began with effusive activity in late December 2020, leading to the formation of a new lava dome. This phase transitioned into an explosive eruption on April 9^th^, 2021, producing powerful ash plumes that rose up to 15–20 kilometers into the atmosphere. These ash clouds spread across several Caribbean islands, severely affecting air quality, aviation, and local ecosystems. The continuous emission of ash and gases during this period created strong atmospheric disturbances, which propagated upward in the form of gravity waves and influenced ionospheric conditions.

We analyzed the TEC variations during the volcanic eruption at Mount La Soufriere from April 9^th^ to April 22^nd^, 2021, with extended observations from March 29^th^ to April 29^th^, 2021, to capture both pre-eruption and post-eruption conditions. The study focuses on the performance of the Hybrid ML-DL Model in comparison with LightGBM, LSTM, MLR and AR models. The eruption period demonstrated significant ionospheric disturbances, particularly during the peak explosive phases, leading to sharp variations and anomalies in TEC. These disturbances created a challenge for accurate TEC prediction, especially for models that rely on linear assumptions and lack the ability to capture complex temporal dependencies.

**Fig 25:**
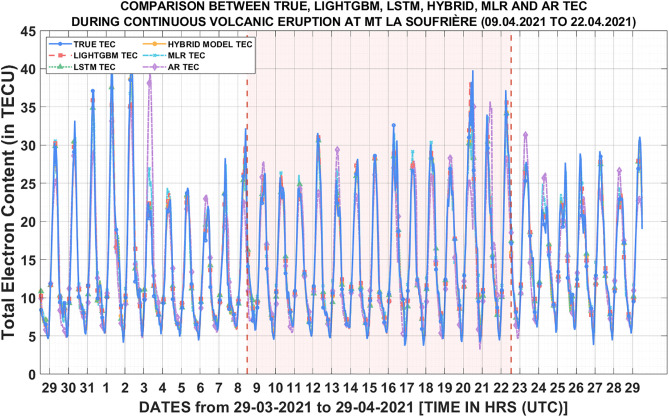
Comparison between TRUE, LightGBM, LSTM, Hybrid ML-DL Model, MLR and AR TEC during volcanic eruption at Mt. La Soufriere (09.04.2021 to 22.04.2021).

Fig 25 illustrates the comparison between True TEC and predicted TEC values obtained from the LightGBM, LSTM, Hybrid ML-DL, and AR models during the volcanic eruption period. The true TEC exhibits strong diurnal variation with peaks reaching approximately 35–40 TECU during daytime hours, particularly during the active eruption phase between April 9^th^ and April 22^nd^.

Notable TEC peaks are observed on April 10^th^ -April 12^th^ and April 19^th^ – April 21^st^, with respect to intensified volcanic activity. The Hybrid model accurately predicts these peaks, while LightGBM slightly underestimates and LSTM produces smoother curves but fails to capture sharp fluctuations. The AR model shows significant deviation, especially during high TEC periods, due to its limitation in modelling non-linear variations. On the whole, the Hybrid ML-DL closely follows the True TEC curve, effectively capturing both periodic variations and sudden TEC variations.

Additionally, abrupt TEC fluctuations are observed during eruption days, especially within short time intervals, which are not fully captured by any model. These deviations are due to sudden variations in volcanic emissions and atmospheric dynamics, introducing complex non-linear effects in the ionosphere.

Table 11 summarizes the performance metrics of the models. From Table 11, we can infer that the Hybrid ML-DL Model’s performance is superior compared to other models. By averaging the performance metrics, the Hybrid model achieved an RMSE of 1.212 TECU, which is lower than 1.462 TECU of LightGBM, 1.822 TECU of LSTM, 1.708 TECU of MLR and 3.355 TECU of AR.

**Table 11:** Performance evaluation and comparison during volcanic eruption at Mt. La Soufriere (29.03.2021 to 29.04.2021).

DATES	RMSE (TECU)					NRMSE					MBD (TECU)					RLE				
	Light GBM	LSTM	Hybrid	AR	MLR	Light GBM	LSTM	Hybrid	AR	MLR	Light GBM	LSTM	Hybrid	AR	MLR	Light GBM	LSTM	Hybrid	AR	MLR
3/29/2021	1.655	1.533	1.306	2.212	1.489	0.064	0.060	0.051	0.086	0.105	0.202	0.378	0.273	0.410	0.286	0.089	0.107	0.078	0.128	0.103
3/30/2021	1.194	1.792	0.928	2.329	1.469	0.042	0.063	0.033	0.082	0.100	0.051	0.462	0.235	0.664	0.192	0.091	0.138	0.066	0.187	0.113
3/31/2021	1.023	1.450	0.850	3.450	2.006	0.032	0.045	0.026	0.107	0.126	0.146	0.275	0.027	1.357	0.260	0.063	0.072	0.048	0.145	0.101
4/1/2021	2.173	2.320	1.813	3.038	2.218	0.060	0.064	0.050	0.084	0.131	0.257	0.937	0.547	1.276	0.578	0.095	0.088	0.068	0.126	0.122
4/2/2021	2.378	2.003	1.263	4.130	2.501	0.063	0.053	0.033	0.109	0.128	0.995	0.271	0.373	2.819	1.052	0.098	0.126	0.063	0.156	0.149
4/3/2021	1.330	2.021	1.264	8.231	2.680	0.076	0.115	0.072	0.468	0.198	0.330	1.354	0.585	4.663	1.556	0.089	0.142	0.082	0.312	0.154
4/4/2021	0.950	1.204	0.799	2.104	1.440	0.054	0.068	0.045	0.119	0.111	0.139	0.684	0.290	0.958	0.948	0.063	0.087	0.051	0.175	0.120
4/5/2021	1.011	2.703	0.911	2.194	1.430	0.052	0.139	0.047	0.112	0.115	0.178	2.186	0.339	1.293	0.208	0.069	0.283	0.064	0.208	0.117
4/6/2021	0.903	2.236	0.888	1.951	1.215	0.051	0.127	0.050	0.111	0.099	0.176	1.233	0.152	1.009	0.142	0.055	0.194	0.054	0.130	0.118
4/7/2021	1.932	1.881	1.628	3.433	2.105	0.085	0.083	0.072	0.152	0.170	0.009	0.672	0.381	0.787	0.537	0.090	0.100	0.070	0.172	0.140
4/8/2021	1.645	1.796	1.363	4.061	2.098	0.063	0.069	0.052	0.156	0.144	0.148	0.370	0.177	1.383	0.412	0.054	0.079	0.044	0.148	0.120
4/9/2021	1.495	1.843	1.309	2.889	1.376	0.073	0.089	0.064	0.140	0.092	0.326	0.087	0.248	0.801	0.017	0.078	0.110	0.060	0.171	0.117
4/10/2021	1.355	1.445	1.094	1.908	1.159	0.064	0.068	0.051	0.090	0.081	0.221	0.406	0.230	0.479	0.648	0.062	0.103	0.051	0.154	0.134
4/11/2021	0.863	1.117	0.617	1.717	1.065	0.047	0.061	0.034	0.094	0.075	0.089	0.005	0.038	0.070	0.287	0.049	0.068	0.037	0.104	0.085
4/12/2021	1.252	1.044	0.915	3.355	1.342	0.047	0.039	0.034	0.126	0.087	0.105	0.002	0.048	1.154	0.246	0.042	0.057	0.031	0.162	0.093
4/13/2021	1.468	1.520	1.223	2.895	1.361	0.071	0.073	0.059	0.140	0.104	0.030	0.151	0.095	1.638	0.762	0.080	0.095	0.068	0.139	0.115
4/14/2021	0.721	1.474	0.627	2.119	1.041	0.033	0.067	0.028	0.096	0.073	0.023	0.640	0.026	1.074	0.043	0.042	0.065	0.033	0.094	0.078
4/15/2021	0.718	0.896	0.558	1.986	0.958	0.030	0.037	0.023	0.083	0.069	0.038	0.268	0.145	0.349	0.230	0.054	0.067	0.038	0.173	0.091
4/16/2021	2.737	2.439	2.403	3.836	2.257	0.101	0.090	0.088	0.141	0.146	0.420	0.391	0.529	1.648	0.412	0.081	0.078	0.061	0.124	0.107
4/17/2021	1.292	1.491	1.024	3.421	1.671	0.056	0.064	0.044	0.147	0.112	0.096	0.070	0.145	0.570	0.533	0.106	0.148	0.089	0.208	0.191
4/18/2021	1.399	1.891	1.252	3.238	1.602	0.053	0.072	0.047	0.123	0.101	0.230	0.790	0.314	1.354	0.118	0.089	0.100	0.078	0.207	0.145
4/19/2021	1.095	1.618	0.915	1.750	1.347	0.049	0.073	0.041	0.078	0.088	0.028	0.560	0.193	0.017	0.212	0.071	0.108	0.059	0.110	0.110
4/20/2021	2.092	3.517	1.992	6.761	4.233	0.059	0.099	0.056	0.190	0.223	0.242	2.084	0.589	4.039	1.693	0.082	0.156	0.077	0.223	0.172
4/21/2021	2.181	3.173	1.850	7.041	2.714	0.073	0.106	0.062	0.236	0.149	0.347	1.345	0.448	1.515	0.294	0.124	0.175	0.104	0.275	0.170
4/22/2021	1.798	2.045	1.193	4.048	1.782	0.055	0.062	0.036	0.123	0.103	0.302	0.467	0.147	0.577	0.107	0.080	0.115	0.061	0.198	0.119
4/23/2021	0.685	1.491	0.540	2.642	1.304	0.031	0.068	0.025	0.121	0.083	0.143	0.719	0.065	1.080	0.551	0.043	0.073	0.032	0.130	0.071
4/24/2021	1.307	2.768	1.152	3.777	2.129	0.082	0.174	0.072	0.238	0.141	0.085	1.050	0.048	0.028	0.371	0.072	0.144	0.053	0.174	0.130
4/25/2021	1.600	1.862	1.282	2.692	1.441	0.072	0.084	0.057	0.121	0.092	0.055	0.229	0.002	0.777	0.044	0.085	0.103	0.069	0.131	0.106
4/26/2021	1.986	2.211	1.500	3.156	1.674	0.086	0.096	0.065	0.137	0.112	0.021	0.007	0.038	0.223	0.424	0.099	0.132	0.083	0.169	0.117
4/27/2021	1.482	1.472	1.156	2.205	1.426	0.058	0.058	0.046	0.087	0.093	0.142	0.078	0.070	0.150	0.121	0.101	0.116	0.081	0.121	0.144
4/28/2021	0.674	0.788	0.473	2.137	1.166	0.035	0.041	0.025	0.112	0.081	0.140	0.472	0.247	0.653	0.268	0.045	0.069	0.034	0.139	0.105
4/29/2021	1.703	1.421	1.101	3.304	1.603	0.066	0.055	0.043	0.128	0.102	0.254	0.141	0.067	0.943	0.076	0.062	0.087	0.046	0.122	0.101
**Total Values**	1.456	1.864	1.160	3.330	1.728	0.062	0.080	0.049	0.137	0.114	0.119	0.459	0.117	0.659	0.426	0.075	0.103	0.061	0.156	0.120
**Average values during volcanic eruption days**	1.462	1.822	1.212	3.355	1.708	0.058	0.071	0.048	0.129	0.107	0.178	0.519	0.228	1.092	0.400	0.074	0.103	0.061	0.167	0.123
**Average values during Non-volcanic eruption days**	1.424	1.883	1.211	3.258	1.744	0.061	0.078	0.049	0.135	0.118	0.291	0.664	0.258	1.279	0.446	0.082	0.119	0.065	0.170	0.118

The NRMSE of the Hybrid model is 0.048, whereas it is 0.058 for LightGBM, 0.071 for LSTM, 0.107 for MLR and 0.129 for AR, indicating the improved prediction accuracy of the Hybrid Model. The MBD values further confirm this, with 0.228 TECU for the Hybrid model, and LightGBM, LSTM, MLR, and AR models showing 0.178 TECU, 0.519 TECU, 0.4 TECU, and 1.092 TECU, respectively. Similarly, the Hybrid model achieved a lower RLE of 0.061, showing superior performance compared to LightGBM (0.074), LSTM (0.103), MLR (0.123) and AR (0.167).

During non-eruption days, all the models demonstrate slight improvement due to stable ionospheric conditions. However, the Hybrid ML-DL Model continues to outperform the other 3 models.

**Fig 26:**
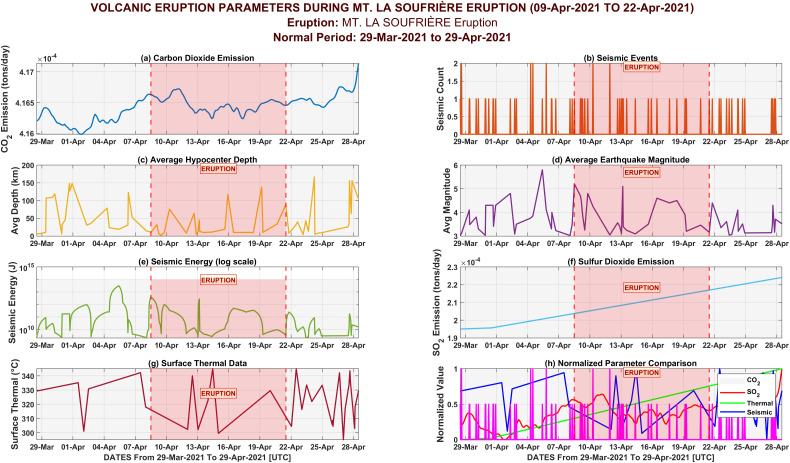
Parameters used during the volcanic eruption at Mt. La Soufriere (09.04.2021 to 22.04.2021).

Fig 26 demonstrates the variation of volcanic parameters such as carbon dioxide emissions, seismic activity, thermal anomalies, and surface deformation during the eruption period. A clear increase in these parameters is observed during the eruption phase, particularly between April 9^th^ and April 22^nd^.

There is a clear indication of a sudden increase in True TEC during the eruption period, particularly around April 10^th^ - April 21^st^. However, the Hybrid ML-DL Model could not fully predict these peak variations due to abrupt and unpredictable changes in volcanic parameters such as seismic activity and gas emissions. Even the AR model fails to capture these sudden peaks due to its linear nature. Similarly, on certain days, including April 11^th^ and April 20^th^, rapid TEC fluctuations were observed, which were not accurately predicted by any model, indicating strong non-linear ionospheric disturbances.

**Fig 27:**
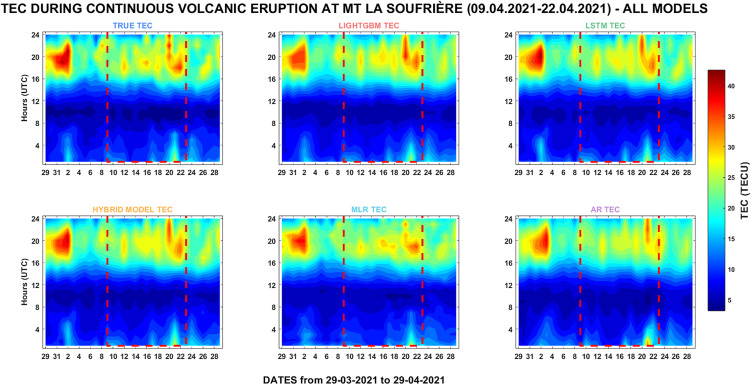
Comparison between TRUE, LightGBM, LSTM, Hybrid ML-DL Model, MLR and AR TEC during volcanic eruption at Mt. La Soufriere (09.04.2021 to 22.04.2021) using Contour plot.

Fig 27 shows the contour plots of true TEC and predicted TEC from all models during the eruption period. The True TEC regions (red/yellow) during daytime and low TEC values (blue) during nighttime.

The Hybrid ML-DL Model contour closely matches the True TEC distribution, accurately replicating both temporal and intensity variations. LightGBM and LSTM moderately match but fail to capture localized high-intensity regions effectively. The AR model exhibits a poor match, with reduced intensity representation and incorrect temporal distribution, showing its inability to capture complex ionospheric behavior.

**Fig 28:**
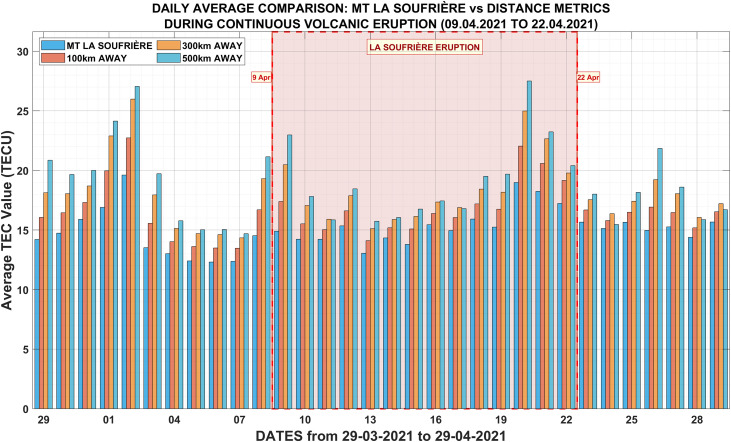
Comparison of daily average TEC during volcanic eruption at Mt. La Soufriere across various distances.

Fig 28 illustrates the daily average TEC values at Mt. La Soufriere and at distances of 100 km, 300 km and 500 km from the volcano, during March 29^th^ to April 29^th^, 2021. The shaded region represents the eruption period (09–22 April 2021). Significant variations in TEC are observed at the volcanic site during this interval, indicating strong ionospheric disturbances caused by the eruption.

At 100 km, noticeable fluctuations remain, showing that the disturbance has also extended to nearby regions. However, with increasing distance, the variations become less effective. At 300 km, the TEC changes are relatively smaller, whereas at 500 km, the values remain nearly stable with minimal variation.

This clearly indicates that the ionospheric impact of the eruption is highly localized and decreases significantly with distance from the source, which results in reduced influence on regions farther away.

### 3.8. Performance evaluation of the Hybrid and Hybrid_combined dataset during the Mt. Ruang volcanic eruption

In order to examine the capability of the proposed framework in forecasting the changes of the ionospheric TEC during volcanic disturbances, two model configurations were tested during the Mt. Ruang volcano eruption period (09 April 2024 to 08 May 2024). The first configuration of the framework was known as the Hybrid model, which was built based on data related to the eruption of Mt. Ruang volcano. The second configuration of the proposed framework was known as Hybrid_Combined, which was constructed by the combination of various volcanic eruptions such as Mt. Kelud (2014), Mt. Sinabung (2014), Mt. Semeru (2021), and Mt. Ruang (2024) data.

Through the evaluation of the performance of the proposed model during the period of Mt. Ruang volcanic eruption by averaging the performance of the model, there was significant variation in the ionosphere TEC. The performance of the Hybrid model was better than that of the Hybrid_Combined model in terms of all statistical metrics. The average RMSE of the Hybrid model is 2.841 TECU during the volcanic eruption day, while the average RMSE of the Hybrid_Combined model is 3.984 TECU. Besides, the NRMSE of the HYBRID model is 0.030, while that of the Hybrid_Combined is 0.043.

Additionally, based on the MBD values, the Hybrid model had a lower average bias of 0.546 TECU, while the Hybrid_Combined model recorded a higher average bias of 0.802 TECU. The Hybrid model also had a lower RLE value of 0.069 relative to 0.106 of the Hybrid_Combined model. These results suggest that the Hybrid model performed better than the Hybrid_Combined model as far as capturing the consistency and reliability in modelling TEC variations due to volcanic activities.

Table 12 summarizes the performance evaluation and comparison of the Hybrid and Hybrid_Combined models during the Mt. Ruang volcanic eruption. In spite of the fact that the Hybrid_Combined model was characterized by the bigger prediction errors, its performance is still important since it takes into account the knowledge obtained from the analysis of other volcanic eruptions with different intensities of eruptions, atmospheric, and ionospheric responses. Thus, the Hybrid_Combined model serves as a basis for assessing the transferability and generalization ability of the proposed framework.

**Table 12:** Performance evaluation and comparison during volcanic eruption at Mt. Ruang (16.04.2024–07-05-2024).

Dates	RMSE(TECU)		NRMSE		MBD(TECU)		RLE	
	Hybrid	Hybrid_ Combined	Hybrid	Hybrid_ Combined	Hybrid	Hybrid_ Combined	Hybrid	Hybrid_ Combined
09-04-2024	2.166	3.093	0.023	0.032	0.297	0.471	0.069	0.063
10-04-2024	2.308	2.874	0.023	0.030	0.322	0.879	0.123	0.092
11-04-2024	2.801	3.239	0.029	0.036	0.475	1.315	0.05	0.080
12-04-2024	3.754	3.888	0.038	0.042	0.56	1.417	0.083	0.168
13-04-2024	3.637	3.567	0.04	0.041	1	0.616	0.048	0.093
14-04-2024	2.321	3.694	0.023	0.038	0.519	0.747	0.047	0.132
15-04-2024	3.267	3.146	0.035	0.033	0.883	0.438	0.048	0.093
16-04-2024	3.546	3.490	0.034	0.034	0.252	0.960	0.103	0.160
17-04-2024	3.666	1.722	0.037	0.019	0.703	0.442	0.062	0.060
18-04-2024	3.819	3.626	0.038	0.035	0.739	0.363	0.059	0.151
19-04-2024	3.74	3.335	0.04	0.035	0.903	0.084	0.05	0.061
20-04-2024	5.952	4.130	0.057	0.042	0.307	0.486	0.1	0.095
21-04-2024	5.047	3.722	0.048	0.040	0.951	0.219	0.088	0.054
22-04-2024	4.498	2.751	0.04	0.027	0.025	0.106	0.084	0.061
23-04-2024	3.467	3.243	0.032	0.035	0.267	0.103	0.065	0.048
24-04-2024	3.104	3.071	0.027	0.029	0.562	0.290	0.061	0.080
25-04-2024	2.949	4.005	0.029	0.043	0.655	0.836	0.038	0.056
26-04-2024	3.183	6.500	0.03	0.063	1.874	1.333	0.129	0.098
27-04-2024	5.111	5.363	0.051	0.051	3.145	0.354	0.147	0.084
28-04-2024	3.861	4.756	0.045	0.042	2.107	0.721	0.093	0.094
29-04-2024	4.159	4.636	0.047	0.042	1.477	1.363	0.174	0.076
30-04-2024	5.097	3.452	0.074	0.030	3.088	0.406	0.151	0.074
01-05-2024	4.45	3.428	0.069	0.034	0.511	0.261	0.089	0.055
02-05-2024	3.916	3.605	0.056	0.034	2.315	1.486	0.104	0.140
03-05-2024	3.834	5.163	0.043	0.052	0.971	0.975	0.263	0.153
04-05-2024	3.058	3.252	0.034	0.038	0.657	0.931	0.069	0.082
05-05-2024	3.084	3.955	0.032	0.045	0.239	0.471	0.076	0.165
06-05-2024	2.472	2.974	0.026	0.043	0.311	1.118	0.082	0.112
07-05-2024	2.972	4.306	0.037	0.067	0.647	1.125	0.074	0.100
08-05-2024	3.929	3.630	0.047	0.051	1.372	1.190	0.122	0.111
Total Values	3.135	3.872	0.040	0.044	0.499	0.806	0.063	0.124
Average Values during volcanic eruption days	2.841	3.984	0.030	0.043	0.546	0.802	0.069	0.106
Average Values during non-volcanic eruption days	2.757	3.301	0.034	0.035	0.132	0.576	0.089	0.094

**Fig 29:**
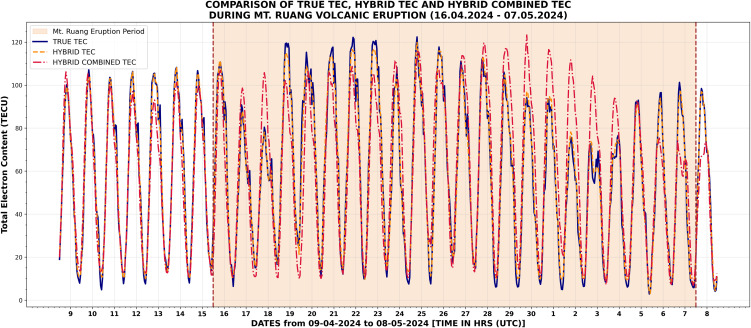
Comparison of True_TEC, Hybrid_TEC and Hybrid_Combined TEC for Mt. Ruang Eruption (16.04.2024- 07.05.2024).

Fig 29 presents the comparison between the observed TEC and the TEC estimated using the Hybrid and Hybrid_Combined models for the Mt. Ruang eruption. The proposed models succeeded in reproducing the temporal variations of TEC; yet, the Hybrid model was more accurate in replicating the observations. For the time periods marked by high activity with fast-increasing and decreasing TEC values, the Hybrid model managed to replicate these disturbances with lower prediction errors compared to the observations. On the contrary, the Hybrid_Combined model showed higher prediction errors due to the overestimation or underestimation of some of the TEC peaks.

In particular, during highly disturbed time periods, the Hybrid model had relatively low prediction errors that indicate its capability of adaptation to the specific ionospheric perturbation caused by the eruption. At the same time, the Hybrid_Combined model demonstrated larger errors in some of the predictions, especially on 26 April 2024, when the RMSE value reached 6.500 TECU in comparison with 3.183 TECU for the HYBRID model. Hence, despite the existence of common signatures in the ionosphere caused by different eruptions, there are unique features of each eruption that can be better predicted by the target-oriented model.

In addition, when comparing the eruptions with the non-eruption days, it is clear that the Hybrid model always showed smaller errors. In non-volcanic days, the Hybrid model produced an average RMSE of 2.757 TECU, while the Hybrid_Combined model had an average RMSE of 3.301 TECU. The average MBD of the Hybrid model and the Hybrid_Combined model was 0.132 TECU and 0.576 TECU, respectively. It can be said that the proposed Hybrid framework has shown high capability not only during ionospheric disturbances but also under relatively quiet conditions.

Finally, it is clear that the eruption-based Hybrid framework produces the best prediction accuracy in the Mt. Ruang eruption case study. On the other hand, the prediction accuracy of the Hybrid_Combined model suggests that the proposed framework still shows reasonable capability of learning generalized TEC response in multiple volcanic eruptions. It can be expected that future studies with larger multi-eruption datasets and leave-one-eruption-out validation strategy will further enhance this capability of the TEC prediction framework for volcanic-ionospheric coupling studies.

## 4. Conclusion

In this research, four prediction methods—Light Gradient Boosting Machine (LightGBM), Long Short-Term Memory (LSTM) networks, a Hybrid ML-DL Model (LightGBM–LSTM Hybrid), and the conventional Multiple Linear Regression (MLR) and Autoregressive (AR) model—were compared for their ability to forecast Total Electron Content (TEC) anomalies in the ionosphere during volcanic eruptions. Six major volcanic events were examined: Mt. Kelud (2014), Mt. Sinabung (2016), Mt. Semeru (2021), Mt. Ruang (2024), Mt. Etna (2013), and Mt. La Soufriere (2021). The selected volcanoes are situated within highly active equatorial zones where the occurrence of instabilities in the ionosphere is common. Volcanic eruptions further make the estimation of TEC very challenging for this region. For model development, GNSS-derived TEC data were used along with volcanic parameters such as CO_2_ and SO_2_ emissions, earthquake counts, thermal anomalies, and surface deformations to develop a better description of the ionosphere’s nonlinear dynamics than what could be achieved with conventional empirical models.

For the Mt. Kelud eruption (from February 13–21, 2014), the RMSE obtained using the Hybrid ML-DL model was 2.699 TECU, which is better than the LightGBM model RMSE value of 3.567 TECU, the LSTM RMSE of 3.873 TECU, the MLR Model RMSE of 5.318 TECU and the RMSE of the AR model of 8.392 TECU. For the NRMSE measure, the Hybrid ML-DL model performed better with 0.036 compared to 0.047 for LightGBM, 0.051 for LSTM,0.109 for the MLR Model and 0.111 for the AR model. MBD values showed that the Hybrid model performed best with 0.382 TECU compared to 1.746 TECU of MLR. For the Mt. Sinabung eruption (May 18–24, 2016), the Hybrid model recorded an RMSE of 1.422 TECU, substantially lower than LightGBM (1.794 TECU), LSTM (2.015 TECU), MLR (2.634) and AR (4.605 TECU), with corresponding NRMSE values of 0.033, 0.041, 0.046, 0.155 and 0.105. During the Mt. Semeru eruption, from December 4–6, 2021, the Hybrid model had an RMSE of 1.132 TECU, whereas LightGBM obtained 1.387 TECU, LSTM was 1.741 TECU, MLR was 2.088 TECU and AR showed 3.903 TECU, and their NRMSE scores were 0.030, 0.037, 0.046, 0.104 and 0.104, respectively. During the extended Mt. Ruang eruption, between April 16 and May 7, 2024, lasting about 22 days and causing TEC perturbations up to 100–110 TECU, the Hybrid model proved its dominance once again. The NRMSE of 0.052 for the Hybrid model compared favorably to 0.06 for LightGBM, 0.084 for LSTM, 0.098 for MLR and 0.141 for AR. For Mt. Etna (November 15–December 2, 2013), the Hybrid model achieved an RMSE of 1.134 TECU against 1.300 TECU for LightGBM, 1.790 TECU for LSTM, 1.953 for MLR and 3.002 TECU for AR. Finally, for Mt. La Soufriere (April 9–22, 2021), in the Hybrid approach, an RMSE value of 1.212 TECU was achieved, which was better than LightGBM (1.462 TECU), LSTM (1.822 TECU), MLR (1.708 TECU) and AR (3.355 TECU), with NRMSE values of 0.048, 0.058, 0.071, 0.107 and 0.129, respectively.

Analysis of ionospheric disturbances with distance from each volcano revealed varying patterns. For Mt. Kelud, where drastic changes in TEC values were seen both at the site of the volcano as well as 100 km from it, these effects had diminished at 300 km while remaining constant at 500 km. For Mt. Sinabung, no drop in TEC enhancement was observed with distance, with disturbances propagating up to 500 km, suggesting a higher degree of volcanic influence on ionospheric perturbations. For Mt. Semeru, noticeable TEC variations were observed at the volcano site and at 100 km, with relatively high values still present at 300 km (20.5–26.0 TECU) and measurable variations at 500 km (19.5–24.0 TECU). For Mt. Ruang, the most dramatic distance propagation was observed, with TEC values at 100 km peaking at 100.0 TECU—almost identical to the volcano site—while values remained consistently high at 300 km (57.0–99.0 TECU) and even at 500 km (55.0–97.0 TECU), demonstrating that ionospheric disturbances from this eruption propagated far into the upper atmosphere with considerable force. For Mt. Etna, volcanic effects decreased sharply with distance, with significant variations at 100 km but relatively constant values at 500 km. For Mt. La Soufriere, noticeable fluctuations remained at 100 km, but variations became smaller at 300 km and nearly stable at 500 km, indicating highly localized ionospheric impacts.

Despite the Hybrid model’s superior performance, certain limitations were observed across all eruption events. On specific dates, abrupt and unpredictable changes in volcanic parameters—including sudden increases in seismic activity, CO_2_ and SO_2_ emissions, thermal anomalies, and surface deformation—caused sudden TEC peaks that neither the Hybrid model nor any other model could accurately predict. For instance, at Mt. Kelud on February 19, 2014, a sudden TEC increase during noon could not be fully captured due to abrupt eruption parameter changes. Similarly, at Mt. Sinabung on May 22, 2016, despite the Hybrid model’s close prediction, the exact peak was missed due to parametric variations. At Mt. Semeru on December 6, 2021, and at Mt. Ruang on April 19, April 29, and May 1, 2024, similar unpredicted peaks occurred due to rapid variations in CO_2_ and SO_2_ emission levels and seismic activity. At Mt. Etna, unexpected spikes occurred on November 23, November 24, December 2, and December 9, 2013, while for Mt. La Soufriere, abrupt TEC changes around April 10–12 and April 19–21 could not be well approximated by any of the models. The AR model performed worst among all tested in each experiment since it is based entirely on previous TEC values through linear dependencies, making the model incapable of predicting TEC changes due to parameter variations caused by volcanic activity.

The current TEC prediction models (LightGBM, LSTM, Hybrid LightGBM-LSTM, AR and MLR), utilized in this study, were built based on temporal data (TEC, seismic data, gas emission, thermal data). The GNSS measurements were not considered from a spatial-topological perspective; hence, correlations among stations were not taken into account when studying atmospheric gravity waves. Thus, we consider these GNSS stations independent of one another with respect to the temporal data used in our study. We agree with you that gravity waves are spatiotemporal phenomena, and that incorporating spatiotemporal relations among GNSS stations could enhance the modelling of gravity waves and their effects on the ionosphere; this could be implemented in the future.

To conclude, machine learning models and, more specifically, the Hybrid ML-DL Model offer remarkable benefits for TEC forecasting in volcanic eruptions. In all six considered cases, the Hybrid model demonstrated the best results in terms of achieving minimal RMSE and NRMSE, minimal bias in MBD, and excellent predictions in RLE. In contrast, the underperforming nature of the AR and MLR model emphasizes the ineffectiveness of pure time series modelling of TEC during volcano-induced disturbances. Extending our research analysis on a bigger dataset and including more deep learning frameworks in the prediction model is one possible future avenue that will further refine the reliability of the prediction system. Doing so will lead to better scientific knowledge about how volcanoes interact with the ionosphere as well as the practical needs of GNSS users in these areas.

## Supporting information

S1 File Dataset. (ZIP)
